# Synthetic anaplerotic modules for the direct synthesis of complex molecules from CO_2_

**DOI:** 10.1038/s41589-022-01179-0

**Published:** 2022-12-05

**Authors:** Christoph Diehl, Patrick D. Gerlinger, Nicole Paczia, Tobias J. Erb

**Affiliations:** 1 Department of Biochemistry and Synthetic Metabolism, Max Planck Institute for Terrestrial Microbiology, Marburg, Germany; 2 Core Facility for Metabolomics and Small Molecule Mass Spectrometry, Max Planck Institute for Terrestrial Microbiology, Marburg, Germany; 3 SYNMIKRO Center for Synthetic Microbiology, Marburg, Germany

**Keywords:** Metabolic engineering, Metabolic pathways, Biocatalysis, Biosynthesis

## Abstract

Anaplerosis is an essential feature of metabolism that allows the continuous operation of natural metabolic networks, such as the citric acid cycle, by constantly replenishing drained intermediates. However, this concept has not been applied to synthetic in vitro metabolic networks, thus far. Here we used anaplerotic strategies to directly access the core sequence of the CETCH cycle, a new-to-nature in vitro CO_2_-fixation pathway that features several C_3_–C_5_ biosynthetic precursors. We drafted four different anaplerotic modules that use CO_2_ to replenish the CETCH cycle’s intermediates and validated our designs by producing 6-deoxyerythronolide B (6-DEB), the C_21_-macrolide backbone of erythromycin. Our best design allowed the carbon-positive synthesis of 6-DEB via 54 enzymatic reactions in vitro at yields comparable to those with isolated 6-DEB polyketide synthase (DEBS). Our work showcases how new-to-nature anaplerotic modules can be designed and tailored to enhance and expand the synthetic capabilities of complex catalytic in vitro reaction networks.

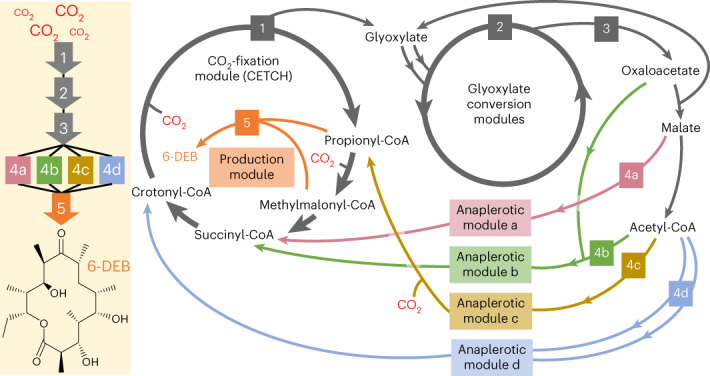

## Abstract (web summary)

Anaplerotic reactions constantly refill metabolic networks with essential intermediates. This concept was adapted to enable a 54-step in vitro biosynthesis of the macrolide backbone of the antibiotic erythromycin from CO_2_.

## Main

Synthetic biology aims at creating biological parts and systems that do not exist in nature. This includes the design and realization of new-to-nature enzymes and metabolic networks that expand the biochemical capabilities of natural metabolism^1,2^. Recent examples are the design and realization of synthetic pathways for the capture and conversion of CO_2_ that are more efficient than natural photosynthesis^3,4^. One of these examples is the CETCH cycle, a synthetic CO_2_-fixing in vitro reaction network that requires 20% less energy compared to the Calvin cycle^5^ and features enoyl-CoA carboxylases/reductases that are an order of magnitude more efficient than Rubisco, the CO_2_-fixing enzyme of photosynthesis^6,7^.

While new-to-nature pathways offer multiple opportunities to access new products and more efficient biosynthetic routes^8–10^, the properties and biosynthetic capabilities of such designer networks still lag behind those of naturally evolved pathways. Natural pathways operate dynamically in the context of living cells and enable the flexible redistribution of metabolic flux depending on the biosynthetic needs of the cell. This is in stark contrast to their synthetic counterparts that are typically limited in metabolic flexibility and adaptability, especially in an in vitro setup^11^.

In the case of the CETCH cycle, one of the shortcomings is that this synthetic pathway is restricted to only one dedicated output reaction that yields the C_2_-compound glyoxylate as a primary CO_2_-fixation product. It has been shown recently that glyoxylate can be further converted into acetyl-CoA to fuel the biosynthesis of different high-value products, including several mono- and sesquiterpenes in vitro^12^. Yet, to harness the full potential of the CETCH cycle, it would be necessary to directly access the cycle’s core sequence intermediates. Being able to directly use different C_3_-, C_4_- and C_5_-CoA thioesters from the CETCH cycle would turn the cycle into a versatile biosynthetic platform that could feed into various biosynthetic routes. However, removing core intermediates from the cycle without refilling them would quickly drain the pool of acceptor molecules required to keep CO_2_ conversion running and inevitably lead to a stalling of the CETCH cycle.

One fundamental building principle of naturally evolved metabolic networks is anaplerosis, that is, reactions or reaction sequences that continuously replenish those intermediates of central carbon metabolism that are directed away into different biosynthetic routes, thereby allowing for continuous and dynamic operation of metabolic networks^13–16^. The defining example is the citric acid cycle, which acts as the turntable of cellular metabolism and is constantly refilled by multiple reaction sequences, such as (phosphoenol) pyruvate carboxylase, malic enzyme and the glyoxylate cycle^17–22^. Consequently, to build synthetic (in vitro) metabolic networks and complex biocatalytic reaction cascades that match the flexibility and adaptability of natural metabolism, it will be essential to include anaplerosis as a fundamental design principle in the design of new-to-nature metabolic systems.

Here we sought to expand the biosynthetic capabilities of the CETCH cycle beyond its output molecule glyoxylate by developing anaplerotic reaction sequences to use inaccessible intermediates from the cycle that would be otherwise inaccessible, in particular propionyl- and methylmalonyl-CoA, which serve as extender units in the biosynthesis of natural products, such as polyketides^23^. Inspired by natural metabolic routes, we designed four anaplerotic reaction sequences for the carbon-neutral and carbon-positive (that is, CO_2_-fixing) conversion of glyoxylate into different intermediates of the CETCH cycle. We reconstructed the different pathways, optimized their performance and tested their ability to support the biosynthesis of the polyketide 6-deoxyerythronolide B (6-DEB)^24,25^, the macrolide backbone of erythromycin directly from CO_2_ via the CETCH cycle.

Overall, implementing the concept of anaplerosis allowed us to establish and operate a complex in vitro metabolic network of more than 50 different enzymatic reactions to continuously synthesize complex molecules from CO_2_ without the need for any additional substrate feeding. Our work represents a stepping-stone toward the realization of biocatalytic cascades mimicking the properties and intricacies of the natural metabolic networks of living cells.

## Results

### Reconstitution of the CETCH cycle (module 1)

**Fig. 1:**
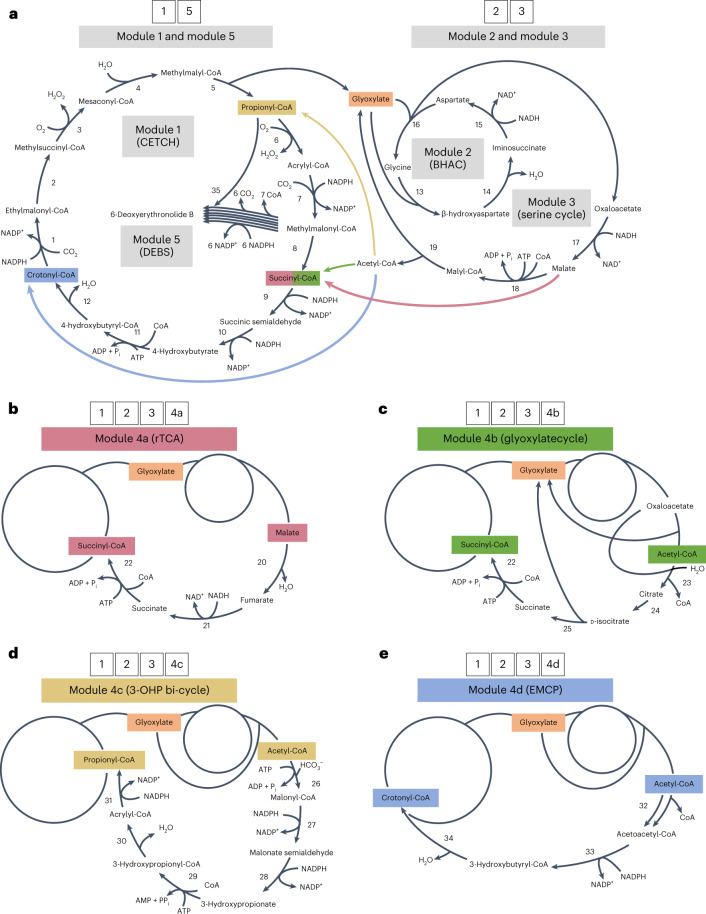
Reaction modules developed in this study. **a**, Core reactions of the CETCH cycle (module 1), β-hydroxyaspartate cycle (BHAC, module 2) and serine cycle (module 3). Module 1 fixes two molecules of CO_2_ per turn and provides one molecule of glyoxylate as output. Modules 2 and 3 convert glyoxylate further into oxaloacetate, malate and acetyl-CoA. The different anaplerotic reaction sequences developed in this study that feed back from module 3 into the CETCH cycle are shown with colored arrows and are further detailed in **b–e**. DEBS (module 5) converts one molecule of propionyl-CoA and six molecules of methylmalonyl-CoA into 6-DEB.

The CETCH cycle revolves around two reductive carboxylation reactions that catalyze two one-carbon extensions (reactions 1 and 7; Fig. 1a). Starting from the C_3_-compound propionyl-CoA, the C_4_-metabolite methylmalonyl-CoA is formed (6 and 7; Fig. 1a) and further converted into the C–-molecule ethylmalonyl-CoA (EMC) through a series of reactions (8–12 and 1; Fig. 1a). EMC is subsequently transformed into methylmalyl-CoA, which is cleaved into glyoxylate and propionyl-CoA (2–5; Fig. 1a). The latter enters another round of the cycle, while the former remains as the primary CO_2_-fixation product and output molecule of the CETCH cycle.

**Fig. 2:**
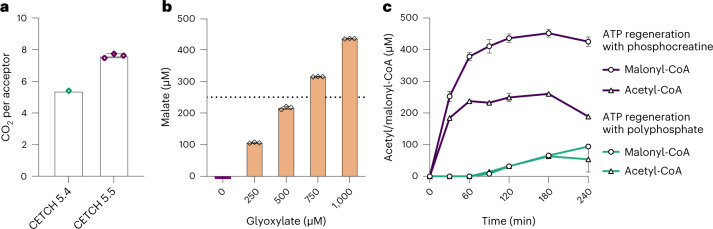
Prototyping and optimization of modules 1–3. **a**, Optimizing the CO_2_-fixation efficiency of module 1 (CETCH cycle). Shown as CO_2_ molecules fixed per molecule of starting substrate (that is, propionyl-CoA) over 2 h for the original CETCH cycle^5^ and the optimized CETCH cycle (CETCH 5.5, this study). In CETCH 5.5, ATP regeneration was changed from a polyphosphate- to a creatine phosphate-based system. **b**, Prototyping module 2. BHAC efficiency (that is, amino group recycling) was tested by providing different glyoxylate concentrations at limiting glycine concentrations. Reactions were started with 0, 250, 500, 750 or 1,000 µM glyoxylate, while the glycine concentration was fixed at 250 µM. Oxaloacetate was converted into malate through Mdh. Reactions were stopped after 60 min, and malate formation was quantified by LC–MS. In the absence of any amino group cycling (that is, if imminosuccinate were completely hydrolyzed into oxaloacetate), malate yield would be limited to 250 µM at glyoxylate concentrations >500 µM (dashed line). The assays with 750 and 1,000 µM glyoxylate indicate almost 90% amino group recycling (Extended Data Fig. 1). **c,** Prototyping and optimization of modules 1–3. Shown are the acetyl-CoA (points) and malonyl-CoA (triangles) yields of combined modules 1–3, when using creatine phosphate-based (purple) or polyphosphate-based (petrol) ATP regeneration. Reactions in **a** and **c** were started with 100 µM propionyl-CoA. Experiments with phosphocreatine-based ATP regeneration included 2 U ml^−1^ creatine phosphokinase. Experiments using polyphosphate-based ATP regeneration used 0.5 U ml^−1^ polyphosphate kinase. Conversion of acetyl-CoA to malonyl-CoA was catalyzed by addition of 100 mU ml^−1^ propionyl-CoA carboxylase D407I (Pcc*, 26; Fig. 1d). All experiments were performed in technical triplicates and are displayed as mean ± s.d., except CETCH 5.4 assays (data were obtained from the original publication^5^). Source data

To establish the CETCH cycle in vitro (module 1; Fig. 1), we modified the setup of recently published version 5.4 (ref. ^5^). To circumvent the use of externally added acetyl-CoA, we replaced the malate readout with a glycolate-based readout, using glyoxylate reductase that irreversibly converts glyoxylate into glycolate. Furthermore, we used a creatine phosphokinase-based ATP regeneration system instead of polyphosphate kinase because high polyphosphate concentrations (≥20 mM) caused precipitations in the assay. The concentrations of individual enzymes (0.4–21 µM) and their respective volumetric activities (0.1–5 U ml^−1^) are listed in Supplementary Table 1. When starting the reaction with 100 µM propionyl-CoA, the modified CETCH cycle produced 381 ± 6.6 µM glycolate within 90 min. This translates into 7.6 ± 0.2 fixed CO_2_ molecules per starting acceptor molecule (propionyl-CoA) and 3.8 cycle turnovers, which is 1.4-fold better than to the original setup of the CETCH cycle (Fig. 2a)^5^.

### Modules 2 and 3: from glyoxylate to central metabolites

To convert glyoxylate, the output molecule of module 1, back into intermediates of the core cycle, we sought to establish downstream reaction modules that would allow us to transform glyoxylate into the central metabolite oxaloacetate, malate or acetyl-CoA, from which several routes into different C_3_- and C_4_-CoA esters exist (see below). To establish these downstream reaction modules, we searched for naturally existing, oxygen-tolerant and carbon-conserving pathway sequences (that is, sequences that do not release CO_2_).

For the formation of oxaloacetate, we aimed at using the β-hydroxyaspartate cycle (BHAC) from *Paracoccus denitrificans* that transforms two molecules of glyoxylate into oxaloacetate through amino-group cycling via the cosubstrate glycine and consumption of one NADH (module 2; Fig. 1a)^12,26,27^. To convert oxaloacetate further, we sought to use reactions of the serine cycle, in particular malate dehydrogenase (Mdh, 17; Fig. 1a), malate thiokinase (Mtk, 18; Fig. 1a) and malyl-CoA lyase (Mcl, 5 and 19; Fig. 1a) that would allow the conversion of oxaloacetate into glyoxylate, which could re-enter the BHAC, and acetyl-CoA (module 3; Fig. 1a)^12^. We reconstituted modules 2 and 3 using volumetric enzyme activities between 0.5 and 8 U ml^−1^ (Supplementary Table 1).

**Extended Data Fig. 1:**
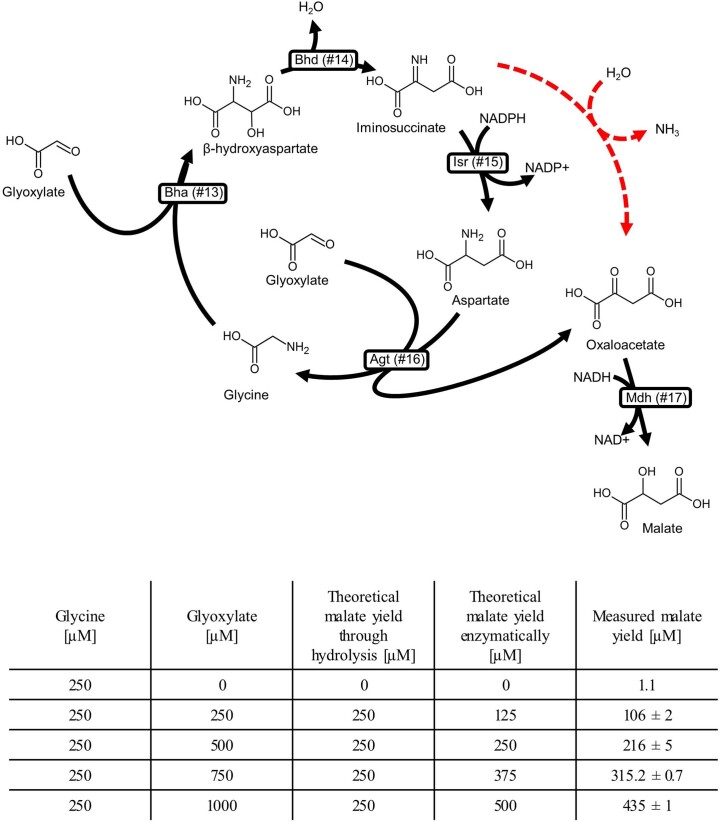
Malate production by the BHAC at varying glyoxylate concentrations. To assess whether the glyoxylate assimilation sequence of the BHAC (reactions #13-17, black arrows) is active, we tested different concentrations of glyoxylate with a set amount of glycine (250 µM). Non-enzymatic hydrolysis (red arrow) of iminosuccinate to oxaloacetate would result in both depletion of the amino-group and oxaloacetate derived from the co-substrate glycine, limiting the detectable yields of malate to equivalent concentration. Thus, malate yields exceeding 250 µM indicate amino-group recycling and multiple turns of the cycle. The results are displayed in the table, and malate concentrations indicating multiple cycle turns were detected for all setups containing a sufficient glyoxylate starting concentration.

One crucial reaction (14, Fig. 1a) in module 2 (that is, the BHAC) is the immediate reduction of the labile intermediate iminosuccinate to prevent the spontaneous hydrolysis of iminosuccinate into oxaloacetate and concomitant loss of the amino group^27^ (Extended Data Fig. 1). Earlier experiments using ^13^C-labeled substrates had shown that an excess of iminosuccinate reductase (Isr, 15, Fig. 1a) over dehydratase (Bhd, #14; Fig. 1a) is necessary to suppress the spontaneous hydrolysis of iminosuccinate and allow the cycle to continuously operate^12^. To test whether a 10-fold excess of Isr (5 U ml^−1^) over Bhd (0.5 U ml^−1^) would be sufficient to drive the BHAC cycle in our setup, we fixed the amino-donor glycine at 250 µM and used different glyoxylate concentrations ranging from 0 to 1,000 µM. To quantify oxaloacetate formation, we used Mdh^28^. In all assays, including those at high (that is, nonlimiting) glyoxylate concentrations, malate yields were close to the expected maximum yield (Fig. 2b and Extended Data Fig. 1), indicating that module 2 indeed turned several times.

### Optimizing the interplay of modules 1–3

Initial coupling of modules 1–3 yielded approximately 100 µM acetyl-CoA, when starting from 100 µM propionyl-CoA and polyphosphate ATP regeneration, indicating one complete turnover through combined modules 1–3 (Fig. 2c). Using a creatine phosphokinase-based ATP regeneration substantially improved the acetyl-CoA yield of modules 1–3 to 260 µM within 3 h. However, acetyl-CoA production had already stalled after 60 min and had even started to decrease at 3 h (Fig. 2c). This was likely caused by the reversible condensation of acetyl-CoA with glyoxylate back into malyl-CoA (5; Fig. 1a) when a certain acetyl-CoA threshold concentration was reached, followed by slow hydrolysis of malyl-CoA over time^5,12,29^.

We aimed at further improving the productivity of coupled modules 1–3 by constantly withdrawing acetyl-CoA through carboxylation into malonyl-CoA, using propionyl-CoA carboxylase variant Pcc* (26; Fig. 1d)^30^, which was described recently^12^. Indeed, the malonyl-CoA yield almost doubled when using Pcc* together with phosphocreatine-based ATP regeneration (Fig. 2c). Reaching a yield of more than 450 µM malonyl-CoA, coupled modules 1–3 were even more productive than module 1 alone, despite their higher demand for energy and reducing equivalents. Starting from 100 µM propionyl-CoA, we observed more than 4.5 cycle turnovers of coupled modules 1–3 within 3 h, and notably, 4 cycle turnovers already after 90 min, compared to only 3.8 cycles for optimized module 1 alone (Fig. 2a). Having established the modules to convert glyoxylate into oxaloacetate, malate or acetyl-CoA, we moved on to the design and implementation of different anaplerotic modules feeding these central intermediates back into the CETCH cycle.

### Design principles of anaplerotic modules 4a–d

In the next step, we drafted different anaplerotic modules to transform oxaloacetate, malate or acetyl-CoA into intermediates of module 1. To that end, we searched for natural pathway segments that would allow us to regenerate the above metabolites into C_3_- or C_4_-CoA esters of the CETCH cycle and were oxygen-tolerant, as well as thermodynamically feasible (that is, show a favorable Gibbs free-energy profile)^5^.

Starting from malate, we identified reactions from the reductive TCA cycle^31^ that produce succinyl-CoA for re-entry into module 1 (module 4a; Fig. 1b). Starting from acetyl-CoA (and oxaloacetate), we identified reactions of the glyoxylate cycle^14^ yielding succinyl-CoA as re-entry point into module 1 (module 4b; Fig. 1c), reactions of the 3-hydroxypropionate (3-OHP)^32,33^ cycle regenerating propionyl-CoA in module 1 (module 4c; Fig. 1d) and reactions of the EMC pathway providing crotonyl-CoA to re-fill module 1 (module 4d; Fig. 1e)^15,34^.

We confirmed the thermodynamic feasibility of anaplerotic modules 4a–d by thermodynamic profiling (Supplementary Fig. 1) and established the different modules in the following. To assess and optimize the different routes, we decided to reconstitute modules 1–3 and 4a–d in vitro, start the reaction with glyoxylate and quantify the production of methylmalonyl-CoA, reasoning that methylmalonyl-CoA levels would serve as a proxy for production yields of 6-DEB, our final benchmark molecule (see below).

### Fumarate reductase levels are important for module 4a

Feedback module 4a (Fig. 1b) is the only C4-conserving pathway that does not start from acetyl-CoA, branching off module 3 after the reduction of oxaloacetate to malate (17; Fig. 1a). Malate is subsequently converted via a fumarate hydratase (Fum, #20; Fig. 1b) into fumarate, reduced to succinate (Frd, 21; Fig. 1b) and finally converted into the core cycle intermediate, succinyl-CoA, by succinate-CoA ligase (Scs, 22; Fig. 1b).

While two of the three additional enzymes required (Fum and Scs) could be directly deployed from *Escherichia* *coli*^35,36^, finding a suitable candidate for the reduction of fumarate proved more difficult. Most Frd enzymes are quinone-dependent multisubunit membrane-bound enzymes, which are oxygen sensitive, excluding their application in an aerobic in vitro setup. However, we identified a suitable candidate from *Trypanosoma brucei* mitochondria, that is, NADH dependent and composed of a single, soluble subunit. Unfortunately, in the absence of fumarate, Frd reduces molecular oxygen, thereby generating H_2_O_2_ and consuming NAD(P)H^37^. While H_2_O_2_ can be detoxified by catalase (Cat), which is present in the assay at 1.5 U ml^−1^, the substrate-independent consumption of NAD(P)H by Frd makes it necessary to fine tune the enzyme’s activity to minimize futile NAD(P)H oxidation.

**Fig. 3:**
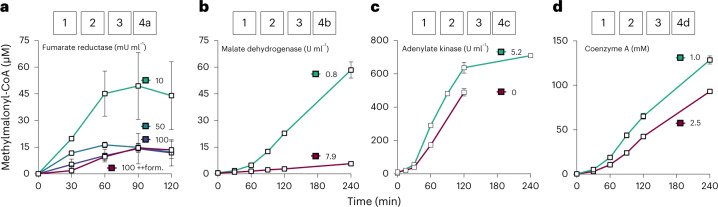
Prototyping and optimization of feedback modules 4a–d. Methylmalonyl-CoA formation from 250 µM glyoxylate through anaplerotic feedback modules 4a–d of varying compositions is presented. All experiments contained modules 1, 2 and 3 together with one of the four anaplerotic modules, in which selected enzymes and/or cofactors were varied. **a**, Module 4a using different amounts of Frd (10, 50 and 100 µU ml^−1^ = 0.01 to 0.1 mU ml^−1^). All experiments contained 50 mM formate, except ++form, which contained 100 mM formate. **b,** Module 4b with different amounts of Mdh. **c,** Module 4c with or without adenylate kinase (Adk). **d**, Module 4d with different amounts of CoA. All experiments were performed in technical triplicates and are displayed as mean ± s.d. Source data

When testing different concentrations of Frd in the context of the full pathway (combined modules 1–3 and 4a) starting from 250 µM glyoxylate, 0.01 mU ml^−1^ Frd resulted in the highest yields of methylmalonyl-CoA (49 ± 18.9 µM after 90 min). Additional formate to increase NAD(P)H regeneration by Fdh did not improve the yield, even when using 0.1 mU ml^−1^ Frd (Fig. 3a). Furthermore, we observed repeatedly high errors in our assays when Frd was added to the master mix. More reliable measurements were obtained when adding Frd just before the start with glyoxylate, and this strategy was adapted for all subsequent assays that included module 4a.

### Mdh controls flux in module 4b

Feedback module 4b (Fig. 1c) resembles the glyoxylate cycle^14^. To synthesize citrate from acetyl-CoA and oxaloacetate (23; Fig. 1c), we decided to use the citrate synthase from *Synechocystis* sp. PCC 6803 (citrate (Cit), 23; Fig. 1c)^38^. In contrast to citrate synthases from heterotrophic bacteria, *Synechocystis* Cit has an ‘inverted’ responsiveness toward allosteric effectors, that is, it shows no inhibition by MgCl_2_, ATP or NADH and becomes activated by ADP. For the generation of isocitrate from citrate (Acn, 24; Fig. 1c), we used AcnA from *E. coli*, which compared to its homolog AcnB has a lower catalytic efficiency but is more oxygen-tolerant^39^. We selected *E. coli* isocitrate lyase to convert isocitrate into glyoxylate and succinate (25; Fig. 1c), which can re-enter the CETCH cycle as succinyl-CoA via succinate-CoA ligase (22; Fig. 1c), as in module 4a.

**Extended Data Fig. 2:**
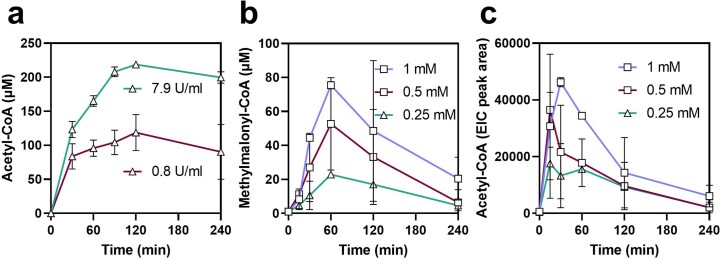
Enzyme and cofactor concentration optimization assays for feedback modules 4b and 4d. **a** Acetyl-CoA accumulation when using module 4b with different amounts of Mdh. More Mdh leads to accumulation of acetyl-CoA and a decrease in methylmalonyl-CoA (see Fig. 3). **b** Methylmalonyl-CoA and **c** acetyl-CoA accumulation when using module 4d with different amounts of CoA (1, 0.5 and 0.25 mM). 1 mM CoA produced the highest concentration of methylmalonyl-CoA and was thus chosen for subsequent experiments. All experiments were performed in technical triplicates and are displayed as mean ± SD. Source data

Note that module 4b uses oxaloacetate (produced by module 2) for the condensation reaction with acetyl-CoA by Cit (23; Fig. 1c) and for the formation of acetyl-CoA itself via module 3 (17–19; Fig. 1a), which leads to a direct competition between Cit and Mdh for oxaloacetate. Due to the more favorable kinetic parameters of Mdh toward oxaloacetate (*K*_m_ ≈ 40 µM, *k*_cat_ ≈ 930 s^−1^)^28^ compared to Cit (*K*_m_ ≈ 90 µM, *k*_cat_ ≈ 2.8 s^−1^, *K*_m-acetyl-CoA_ = 220 μM)^38^, we hypothesized that a successful implementation of module 4b would directly depend on the concentration of Mdh. Indeed, the amount of acetyl-CoA produced when combining modules 1, 2, 3 and 4b showed an inverse correlation with the amount of Mdh used (Extended Data Fig. 2a). Using 7.9 U ml^−1^ Mdh caused an accumulation of acetyl-CoA and produced only 6 µM methylmalonyl-CoA, indicating that the oxaloacetate pool was completely drained by Mdh (Fig. 3 and Extended Data Fig. 2a). In contrast, combined modules 1–3 and 4b with 0.8 U ml^−1^ Mdh produced almost 60 µM methylmalonyl-CoA within 2 h, indicating that sufficient oxaloacetate was available for the Cit reaction. Therefore, we chose this Mdh concentration for the implementation of module 4b.

### Improving module 4c through adenylate kinase

Feedback module 4c (Fig. 1d) is based on the reactions of the 3-OHP bi-cycle. We used the aforementioned engineered propionyl-CoA carboxylase (Pcc*, 26; Fig. 1d)^30^ to produce malonyl-CoA from acetyl-CoA. Malonyl-CoA is further reduced via a bifunctional malonyl-CoA/malonate semialdehyde reductase (27 and 28; Fig. 1d)^40,41^, resulting in 3-OHP. Finally, for the reaction of 3-OHP to propionyl-CoA (29–31; Fig. 1d), we used propionyl-CoA synthetase (Pcs), a multicatalytic nanocompartment that was characterized recently^42^. Module 4c comprises of only three enzymes catalyzing six reactions that consume three NADPH and three ATP equivalents and shows the strongest thermodynamic driving force compared to the other feedback modules (Supplementary Fig. 1). Initial experiments resulted in the formation of up to 490 µM methylmalonyl-CoA after 2 h starting from 250 µM glyoxylate (Fig. 3c). The addition of Adk to regenerate the AMP produced by Pcs increased the yield by 30% after 2 h and led to the formation of more than 700 µM methylmalonyl-CoA by combined modules 1–3 and 4c after 4 h (Fig. 3c).

### Productivity of module 4d is controlled by CoA

Feedback module 4d comprises a partial EMC pathway (Fig. 1e). This module requires only three reactions to yield crotonyl-CoA that re-enters the CETCH cycle before the carboxylation step (1; Fig. 1a). Module 4d requires no ATP and one NADPH and has the lowest thermodynamic driving force (Supplementary Fig. 1).

**Extended Data Fig. 3:**
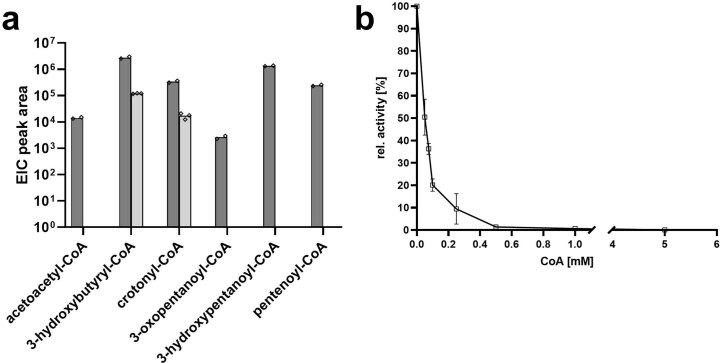
Aat promiscuity and inhibition of the condensation reaction by free Coenzyme A. **a** Promiscuity of Aat assayed with acetyl-CoA and propionyl-CoA. Dark gray bars display EIC peak areas obtained for each product of condensation, reduction and dehydration reactions in an assay containing all three pathway enzymes (Aat, Aar, Bbd), acetyl-CoA and propionyl-CoA. All products for acetyl- and propionyl-CoA combinations were detectable, in contrast to the condensation of two propionyl-CoA. When combining modules 1-2-3-4d, only 3-hydroxybutyryl-CoA and crotonyl-CoA were detected (light gray). Measurements were performed in technical duplicates and are displayed as mean. **b** Aat activity coupled to Aar with varying amounts of free CoA. Obtained activities were set relative to the measurement containing no additional free CoA. To ensure Aar was not inhibited by free CoA, a control where CoA was added to the Aat reaction after 20 minutes followed by addition of Aar, was performed and showed no differences to the corresponding setup using all enzymes (data not shown). Measurements were performed in technical triplicates and are displayed as mean ± SD. Source data

To establish the pathway, we used acetyl-CoA acetyltransferase (Aat, 32; Fig. 1e), its cognate reductase Aar (33; Fig. 1e)^43^ and a putative β-hydroxybutyryl-CoA dehydratase (Bbd, 34; Fig. 1e) that we characterized in more detail (Extended Data Fig. 3 and Supplementary Fig. 2). Aat showed some eveidence of a promiscuous side reaction with propionyl-CoA in vitro (Extended Data Fig. 3a). However, when testing module 4d together with modules 1–3, we did not observe the formation of side products (Extended Data Fig. 3a), indicating that Aat promiscuity was not relevant in the context of the full system. The condensation reaction of Aat is inhibited by free CoA^44^ (Extended Data Fig. 3b), which we use at a 1 mM concentration in the CETCH cycle. To assess the effects of free CoA on the context of the full system, we systematically varied the initial amount of free CoA up to 1 mM in the assay with combined modules 1–3 and 4d starting from 250 µM glyoxylate. Using 1 mM CoA in the assay caused a strong accumulation of acetyl-CoA compared to lower CoA concentrations (Extended Data Fig. 2c), which is in line with the observation that isolated Aat was almost completely inhibited by 1 mM CoA (Extended Data Fig. 3b). Interestingly, however, 1 mM CoA also resulted in the highest methylmalonyl-CoA yields, indicating that productivity of the whole system was not negatively affected through CoA (Extended Data Fig. 2b). To test the influence of even further increased CoA levels to yield, we repeated the experiment with 1 and 2.5 mM CoA. Using 2.5 mM CoA reduced methylmalonyl-CoA formation to 93 µM, compared to the setup with 1 mM (Fig. 3d; 129 µM), indicating that the standard setup of 1 mM free CoA in module 1 was suited for operating combined modules 1–3 and 4d.

### Anaplerotic feedbacks enable 6-DEB production from CO_2_

**Extended Data Fig. 4:**
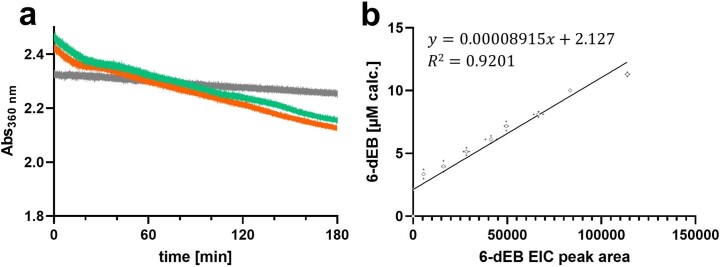
6-dEB quantification. 6-dEB formation was quantified by NADPH oxidation (6 NADPH molecules per 6-dEB molecule). This data was used to calibrate HPLC-MS measurements (EIC peak area). **a** Spectrophotometric NADPH oxidation of DEBS (in duplicates; green and orange) and a negative control without substrates (gray). **b** After establishing stoichiometric equivalence between NADPH oxidation and 6-dEB production, the reduction rates were correlated to the 6-dEB HPLC measurements in increments of 20 minutes, performed as technical duplicates. Note that the ordinate offset has been subtracted to yield the concentrations given in the main text. Source data

To test our anaplerotic modules in a complex biosynthetic scenario, we aimed at applying coupled modules 1–3 plus either module 4a, 4b, 4c or 4d for the production of 6-DEB. 6-DEB is synthesized by DEBS, a type I polyketide synthase (PKS) that uses propionyl-CoA as a starter unit and six methylmalonyl-CoA equivalents as extender units per molecule of 6-DEB (module 5; Fig. 1a and Supplementary Fig. 3). 6-DEB is synthesized via six subsequent decarboxylative Claisen condensations, accompanied by CoA-release and consumption of six NADPH^45^. To measure 6-DEB, we followed an established high-performance liquid chromatography–mass spectrometry (HPLC–MS)-based method that we calibrated with self-produced 6-DEB standards (Methods; Extended Data Fig. 4)^25^.

**Fig. 4:**
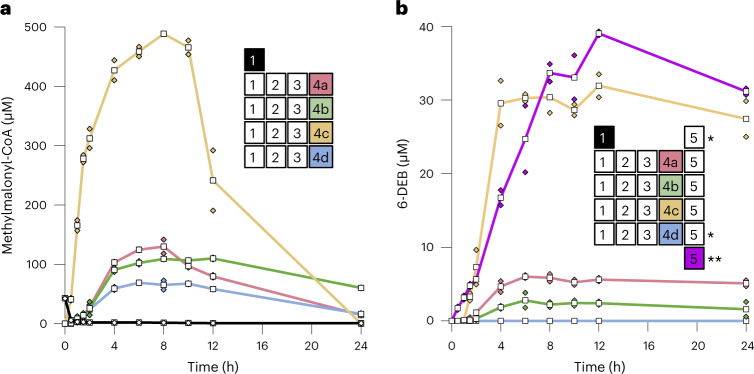
Prototyping and application of the different anaplerotic pathways for 6-DEB production. **a**, Methylmalonyl-CoA formation of the different anaplerotic modules 4a, 4b, 4c, or 4d when coupled to modules 1–3. Experiments were started with 250 µM glyoxylate (coupled modules 1, 2, 3 plus either module 4a, 4b, 4c or 4d) or 167 µM propionyl-CoA (module 1 alone). **b,** 6-DEB formation by the different anaplerotic pathways modules 4a, 4b, 4c or 4d when coupled to modules 1–3 and DEBS (module 5). Shown also is the 6-DEB formation of module 1 coupled to module 5 without any anaplerotic modules, and module 5 alone, started with propionyl-CoA and methylmalonyl-CoA. Assays were started using either 167 or 333 µM propionyl-CoA. For details and additional data, see Extended Data Fig. 5. *, no 6-DEB detected. **, started with 0.8 mM propionyl-CoA and 1.0 mM methylmalonyl-CoA. All experiments were performed in technical duplicates and are displayed as mean (white squares) of the individual measurements (colored squares). Source data

**Extended Data Fig. 5:**
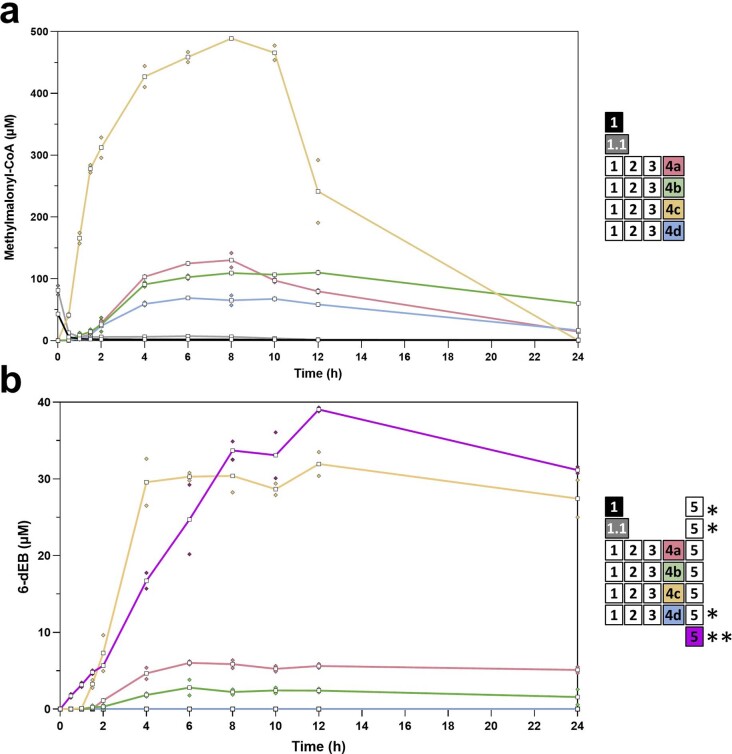
Extended version of Fig. 4a,b. Setups containing anaplerotic sequences (1-2-3-4[a/b/c/d]) were started with 250 µM glyoxylate (C2). Module 1 alone was started with either an equivalent amount of propionyl-CoA (C3, 167 µM) or twice the amount (333 µM, labeled as 1.1). a Methylmalonyl-CoA accumulation in the different feedback pathways and both setups of module 1 alone. Note that the 0 h samples for both setup 1 and 1.1 contained detectable amounts of methylmalonyl-CoA due to a fast turnover immediately after addition of starting substrate (propionyl-CoA). b 6-dEB production by the different pathways and a control using only the isolated polyketide synthase. *not detectable **positive control was started with 0.8 mM propionyl-CoA and 1.0 mM methylmalonyl-CoA. All experiments were performed in technical duplicates and are displayed as mean (white squares) and their individual data points (colored squares). Source data

Before quantifying 6-DEB production, we first verified and compared the methylmalonyl-CoA production of our four optimized pathways without module 5 (that is, without DEBS). When testing coupled modules 1–3 plus either module 4a, 4b, 4c or 4d, all four feedback routes accumulated methylmalonyl-CoA at levels comparable to those observed earlier (Figs. 3 and 4a). Peaking at around 8 h, the different anaplerotic pathways converted 250 µM glyoxylate into methylmalonyl-CoA with effective carbon conversions (that is, carbon percentage yield of methylmalonyl-CoA over starting substrate) at approximately 110% (modules 1–3 and 4a), 90% (modules 1–3 and 4b), 400% (modules 1–3 and 4c) and 60% (modules 1–3 and 4d; Fig. 4a, Extended Data Fig. 5 and Supplementary Table 2). Thus, two of the four anaplerotic pathways were carbon positive (that is, incorporated additional CO_2_ into methylmalonyl-CoA) under these conditions. The observed decrease of methylmalonyl-CoA after 8 h was likely caused by further conversion and/or hydrolysis of the CoA ester in the assay.

**Extended Data Fig. 6:**
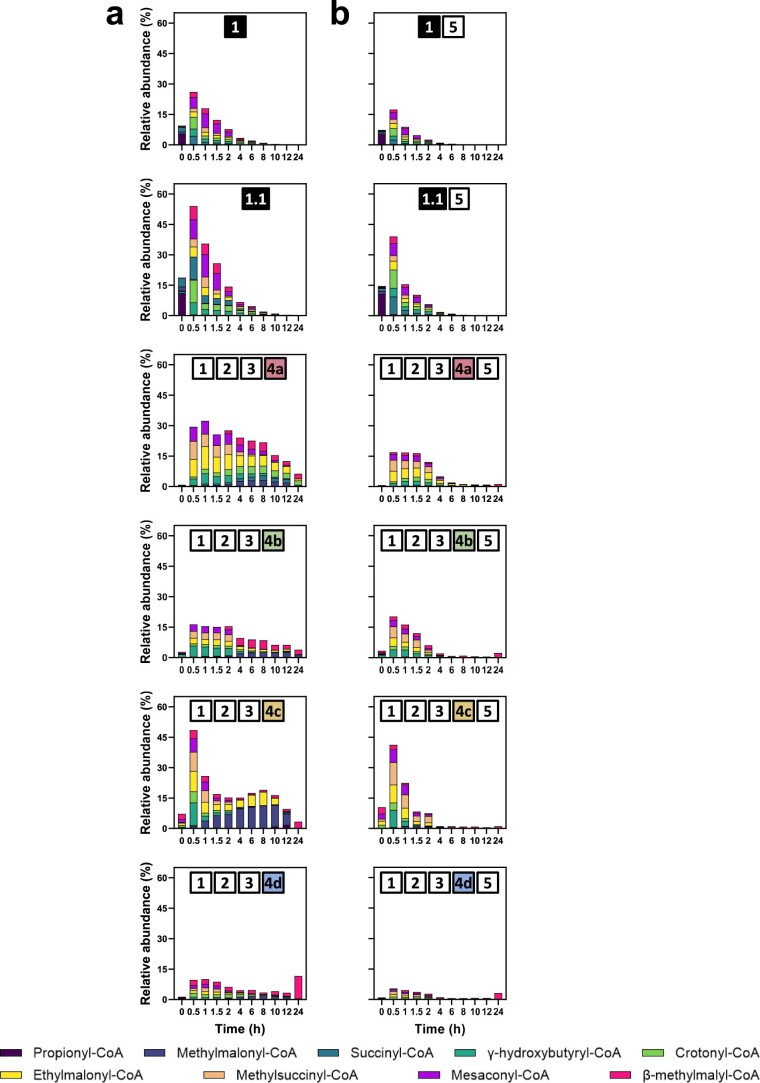
Relative accumulation of CoA intermediates in the feedback systems with and without module 5. All left panels (**a**) show relative module 1 intermediate accumulation over time without module 5 (DEBS), right panels show intermediate accumulation with module 5 (**b**). Addition of module 5 had a consistently suppressive effect on the accumulation of intermediates compared to the setups without. The highest detectable extracted ion count via HPLC-MS (as EIC peak areas) measurement of each individual CoA thioester in any assay shown in Fig. 4 was set to 11.11% (9 compounds = 100%). All other EIC peak areas of that compound were set relative to that to determine the relative abundance. For absolute values of the EIC peak areas see Supplementary Fig. 4. Setups containing only module 1 (± module 5) were started with propionyl-CoA (167 µM in 1, and 333 µM in 1.1), all others with 250 µM glyoxylate. Source data

**Extended Data Fig. 7:**
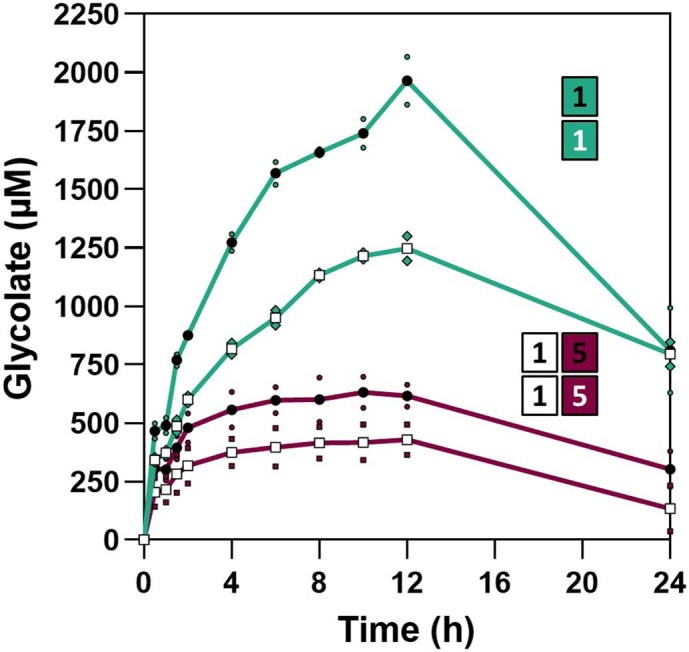
Glycolate production of module 1 in the presence or absence of module 5. Glycolate production of module 1 (CETCH core cycle) with and without module 5 (DEBS) shows that glycolate yield is reduced by almost 70% by addition of module 5. The assays are technical duplicates and were either started using 167 µM propionyl-CoA (white squares) or 333 µM propionyl-CoA (black circles). All experiments were performed in technical duplicates and are displayed as mean and their individual data points. Source data

When testing different pathways together with DEBS (coupled modules 1–3, plus either module 4a, 4b, 4c or 4d, and module 5), three of the four anaplerotic modules yielded detectable amounts of 6-DEB starting from glyoxylate (Fig. 4b and Extended Data Fig. 5). In contrast, the ‘control’ (that is, coupled modules 1 and 5 without anaplerotic feedback) did not produce detectable amounts of 6-DEB, when starting from 167 or 333 µM propionyl-CoA. Notably, addition of DEBS (module 5) to module 1 changed the CoA ester profile of the CETCH cycle (compare module 1 with combined modules 1 and 5, Extended Data Fig. 6 and Supplementary Fig. 4) and reduced the glycolate yield by almost 70% (compare module 1 with combined modules 1 and 5, Extended Data Fig. 7), indicating that module 1 and 5 were negatively affecting each other without a functional anaplerotic feedback.

Surprisingly, the setup with feedback module 4d did also not show detectable polyketide production, even though modules 1–3 and 4d had produced methylmalonyl-CoA at relevant concentrations in the absence of module 5 before (Fig. 4a)^46^. In the presence of module 5, the CoA ester profile of modules 1–3, 4d and 5 was changed, indicating that DEBS negatively influenced carbon flux through the system, probably by depleting the system quickly of propionyl- and/or methylmalonyl-CoA (compare combined modules 1–3 and 4d with combined modules 1–3, 4d and 5, Extended Data Fig. 6 and Supplementary Fig. 4), similar to the effect observed in the ‘control’ (see above).

The setups with feedback modules 4a or 4b yielded 6.0 ± 0.2 µM and 2.8 ± 1.0 µM 6-DEB, respectively, while the setup with module 4c produced 31.9 ± 1.6 µM 6-DEB (Fig. 4b). Synthesis of one molecule of 6-DEB requires one C_3_-CoA (propionyl-CoA) and six C_4_-CoAs (methylmalonyl-CoA). Thus, the production of 32 µM 6-DEB through combined modules 1–3, 4c and 5 was carbon positive, with an effective carbon conversion of 172% (Supplementary Table 2), suggesting that combined modules 1, 2, 3, 4c and 5 had successfully transformed CO_2_ into 6-DEB. Notably, combined modules 1–3, 4c and 5 starting with 0.25 mM of the C_2_-substrate glyoxylate produced 6-DEB (31.9 ± 1.6 µM) at yields comparable to that obtained with the isolated PKS alone (module 5, 39.1 ± 0.3 µM) provided with 1.8 mM C_3_- and C_4_-building blocks (that is, 0.8 mM propionyl-CoA and 1 mM methylmalonyl-CoA; Fig. 4b). Overall, these data did not only demonstrate the direct conversion of CO_2_ into 6-DEB through more than 50 in vitro reactions but also showed that our reaction network was able to operate with similar activity as the isolated DEBS PKS, highlighting the importance and potential of anaplerotic feedback systems for establishing continuously operating in vitro reaction networks and biocatalytic cascades.

## Discussion

One of the biggest challenges in contemporary biology and chemistry is to construct synthetic systems that exhibit the complexity and characteristics of naturally existing biological systems. Here we aimed to create an in vitro catalytic network that is, able to produce a chemically challenging molecule, 6-DEB, directly from CO_2_. This was achieved by developing and subsequently coupling different reaction modules, and in particular different anaplerotic reaction sequences, to replenish core metabolites of the network by CO_2_. While the core network alone (CETCH cycle, module 1) failed to synthesize 6-DEB, our anaplerotic feedback modules enabled it to produce the polyketide with up to 172% carbon conversion and at yields that are comparable to that with the isolated polyketide machinery in vitro. Our results highlight the importance of anaplerotic reaction sequences, not only for the continuous operation of natural metabolism but also for complex synthetic catalytic networks. While we focused our work on 6-DEB as a model product, we note that many other complex molecules, such as other polyketides and polymers (polyhydroxyalkanoates), could in principle be derived from the core cycle augmented with our anaplerotic modules.

What determines the productivity of anaplerotic reaction modules? One important aspect seems to be their thermodynamic driving force, that is, their Gibbs free-energy profile (Supplementary Fig. 1; module 4c > 4b > 4a > 4d) but likely also the re-entry point of the different modules into the core network (CETCH cycle, module 1). Module 4c that shows the highest thermodynamic driving force directly yields propionyl-CoA, the starter unit of DEBS, which can be converted by just two additional steps into methylmalonyl-CoA, the extender unit of DEBS. In contrast to module 4c, modules 4a and 4b yield succinyl-CoA and, therefore, require another 11 and 13 enzymatic reactions, respectively, to arrive at the same metabolites, and are potentially subject to more kinetic and enzymatic bottlenecks. As an example, Frd of module 4a has a high side reactivity with NAD(P)H and oxygen as an alternative electron acceptor, depleting NAD(P)H pools over time. This likely affects the activity of all NAD(P)H-dependent enzymes in the reaction network, which is indicated by accumulation of reaction substrates of NAD(P)H-dependent enzymes in combined modules 1–3, 4a and 5 (for example, succinyl-CoA and crotonyl-CoA; Supplementary Fig. 4c and 4e). Further protein engineering efforts or screening of alternative homologs could yield variants overcoming the deficiencies of Frd or any other enzymes constraining the reaction network. Alternatively, NAD(P)H depletion in module 4a may also be addressed by dynamically responding to cofactor purge valves, as described recently in the context of an in vitro PHA biosynthetic system^47^.

Note that such a cofactor valve might further explain the efficiency of module 4c. Pcc* in feedback module 4c not only catalyze the carboxylation of acetyl-CoA into malonyl-CoA but also is still able to carboxylate propionyl-CoA into methylmalonyl-CoA^30,48^. Thus, two different routes to yield methylmalonyl-CoA can operate in parallel in module 4c, one ATP dependent and the other requiring NADPH. This might allow module 4c to dynamically react to cofactor availability by alternating between pathways for the propionyl-CoA to methylmalonyl-CoA conversion, similar to a molecular rheostat^49^.

Finally, balancing and maintaining propionyl- and methylmalonyl-CoA pools seem to have a particularly important role for network productivity, especially when considering how DEBS influences flux through the metabolic network (Extended Data Fig. 6 and Supplementary Fig. 4). Addition of DEBS to module 1 decreased glyoxylate formation, indicating that draining metabolites from the CETCH cycle effectively stalls module 1 (Extended Data Fig. 7), which further underlines the importance of anaplerotic modules to keep the CETCH cycle turning and allow for continuous 6-DEB synthesis at the same time.

Overall, the successful coupling and simultaneous operation of up to 54 reactions provide a first step toward the development of dynamic in vitro catalytic networks. While our work demonstrates that anaplerotic reaction sequences provide more flexibility to catalytic networks, more and additional layers of regulation will be required to build complex catalytic systems that show the intricate design of natural metabolic networks. This includes the allosteric control and/or compartmentalization of reactions, as well as layers of translational regulation to dynamically regulate catalytic networks. Approaches using cell-free transcription-translation systems and recent efforts to couple synthetic metabolism to light-controlled energy modules might provide the requirements to establish such exquisite control in the future, paving the way for further efforts that make use of complex enzymatic cascades in biology and chemistry^50^.

## Methods

### Plasmid construction

Except for Cit, all plasmids used were constructed previously (Supplementary Table 3). For the construction of a Cit expression plasmid, previously described methods were adapted^38^. In brief, *Synechocystis* sp. PCC 6803 was grown in BG-11, collected via centrifugation and lysed through sonication and the cell debris was used as a template for PCR amplification of its citrate synthase with the following primers: forward, CAAGGTACCGACTGATAACGAAGTGTTTAAAG; reverse, CTGCGGCCGCTTAAATAATCGCATTGGGGTC. The corresponding product was purified and, together with the target vector pET-51b, cut with FastDigest restriction enzymes (Thermo Scientific) KpnI and NdeI (sited underlined). Following DNA purification, vector and insert were ligated with a T4 ligase according to protocol (New England Biolabs, NEB) and the ligation mix was transformed into electrocompetent *E. coli* DH5-α. The final construct (N-Strep Cit) was verified via sequencing (Microsynth).

### Production and purification of recombinant proteins

Unless otherwise denoted, all proteins described in Supplementary Table 3 were purified as described in the following. After transformation of *E. coli* expression strains (*E. coli* BL21(DE3) Rosetta (Novagen) for Mco and Pco expression; *E. coli* BAP1 (ref. ^51^) for production of DEBS proteins; *E. coli* BL21(DE3) (Thermo Scientific) carrying an additional plasmid co-expressing GroEL and GroES chaperones for Hbs production; *E. coli* BL21(DE3) (Thermo Scientific) for expression of all other proteins), 2 L of salt-buffered TB medium was directly inoculated with colonies from the selection plates and grown at 37 °C and 90 r.p.m. till OD_600_ of 0.5–1.0 was reached. Subsequently, cultures were cooled down to 21 °C, induced with 25 µM IPTG and grown overnight. For cultures producing Hbd, 100 µM Fe(II)SO_4_, 100 µM Fe(III)citrate and 20 mM fumarate If not already included were added at induction, grown to an OD_600_ of 4 and cooled down in a sterile Schott bottle for protein production under microaerobic conditions. Furthermore, the production of Pco was done at 25 °C for 4 h. Following cell pellet collection by centrifugation (15 min, 4 °C, 6,000*g*), the cells were resuspended in two parts (wt/vol) lysis buffer (buffer A, 500 mM NaCl, 50 mM HEPES, 10% glycerol, pH 7.8) and 5 mM MgCl_2_, 10 µg ml^−1^ DNase and one tablet of SigmaFAST Protease Inhibitor Cocktail (Sigma-Aldrich) was added. Cell lysis was performed using a microfluidizer (two iterations at 16,000 psi), followed by centrifugation at 50,000*g* for 1 h at 4 °C. The supernatant was filtered through a 0.45 µm membrane, mixed with 3 ml preequilibrated (buffer A) Protino Ni-NTA agarose beads (Macherey-Nagel) and incubated on ice for 30–45 min (70 r.p.m.). Afterward, the beads were collected in a 14 ml gravity column and washed with three column volumes (CVs) of lysis buffer, followed by two washing steps with three CVs of slysis buffer containing additional 50 mM of imidazole and three CVs with 75 mM imidazole. Elution was done with two CVs of lysis buffer containing 500 mM imidazole (buffer B). The elution fractions were concentrated with an Amicon Ultra 15 ml Centrifugal Filters (Merck), possessing an adequate molecular weight cutoff.

All CETCH core enzymes and the propionyl-CoA synthase were desalted on a HiLoad 16/600 Superdex 200-pg column (GE Healthcare). Downstream enzymes for the feedback modules 1, 2a, 2b and 2c were desalted with 2 × 5 ml HiTrap desalting columns (GE Healthcare). For both steps, a desalting/storage buffer containing 200 mM NaCl, 50 mM HEPES and 10% glycerol at pH 7.8 (buffer C) was used. For Hbs and Hbd, buffer C contained 500 mM NaCl. All DEBS proteins, except the strep-tagged LD(4) (see below), were additionally separated using a 5-ml Q-Sepharose HiTrap anion exchange column (GE Healthcare), with an 80 ml gradient from buffer D (50 mM HEPES, 10% glycerol, pH 7.8) to buffer E (500 mM NaCl, 20% glycerol, pH 7.8). The collected fractions were pooled and concentrated again. FAD was added to Pco and Mco equivalent to the protein concentration. Enzymes requiring MgCl_2_ or coenzyme B_12_ were stored in buffer C containing 2 mM of the respective cofactor. If not already included in the storage buffer, glycerol was added to a final concentration of 20% (vol/vol) and the proteins were flash-frozen in liquid nitrogen and stored at −80 °C.

Production of proteins containing a Strep-Tag (LD(4) and Cit) was as stated above. Buffer C was used for all the following steps. After lysis and centrifugation, the supernatant was loaded onto a preequilibrated 1-ml StrepTrap column (GE Healthcare) and ultimately eluted using buffer C containing 2 mM d-desthiobiotin. Concentration and storage did not differ from the steps described above.

### Enzyme assays

All samples were processed as follows: the samples were taken and quenched in a solution containing 10% (vol/vol) 50% formic acid and 10% (vol/vol) 500 mM sodium polyphosphate to promote protein precipitation, except for the coupling experiments (Fig. 4) where no polyphosphate was used. The samples were centrifuged at 11,000*g* for 20 min at 4 °C to pellet the proteins. The supernatant was transferred into a new tube and stored at −80 °C until measurement.

#### CETCH 5.5 (Fig. 2a)

The assay for CETCH 5.5 was in triplicates in an assay volume of 30 µl and included 100 mM HEPES-KOH pH 7.5, 5 mM MgCl_2_, 20 mM phosphocreatine, 50 mM bicarbonate (NaHCO_3_), 20 mM formate (HCOONa), 1 mM CoA, 0.1 mM coenzyme B_12_, 2 mM ATP and 5 mM NADPH. The enzyme were used at the concentrations shown Supplementary Table 1. The reaction was started with 100 µM propionyl-CoA, and the tubes were shaken at 450 r.p.m. at 30 °C. Samples (8 µl) were taken after 90 min and quenched.

#### BHAC validation (Fig. 2b)

The assay for BHAC validation was in triplicates in an assay volume pf 50 µl and included 100 mM HEPES-KOH pH 7.5, 5 mM MgCl_2_, 10 mM NADPH, 10 mM NADH, 0.25 mM glycine and 0.1 mM pyridoxalphosphat. The enzymes for BHAC and Mdh were used according to the concentrations shown in Supplementary Table 1. The reactions were started with 0, 0.25, 0.5, 0.75 or 1 mM of glyoxylate, and the tubes were shaken at 450 r.p.m. at 30 °C. Samples (12 µl) were taken after 60 min and quenched.

#### CETCH to acetyl-CoA optimization (Fig. 2c)

The assay was performed in triplicates in an assay volume of 100 µl and oincluded 100 mM HEPES-KOH pH 7.5, 5 mM MgCl_2_, 20 mM phosphocreatine or sodium polyphosphate, 50 mM bicarbonate (NaHCO_3_), 20 mM formate (HCOONa), 1 mM CoA A, 0.1 mM coenzyme B_12_, 5 mM ATP, 5 mM NADH, 5 mM NADPH, 1 mM glycine and 0.1 mM pyridoxalphosphat. The enzymes were used at the concentrations shown in Supplementary Table 1. One setup was done with and one without Pcc*. The reactions were started with 100 µM propionyl-CoA, and the tubes were shaken at 450 r.p.m. at 30 °C. Samples (12 µl) were taken at 0, 30, 60, 90, 120, 180 and 240 min and quenched.

#### CETCH with module 4a (Fig. 3a)

The assay was performed in triplicates in an assay volume of 50 µl and included 100 mM HEPES-KOH pH 7.5, 10 mM MgCl_2_, 20 mM phosphocreatine, 50 mM bicarbonate (NaHCO_3_), 50 or 100 mM formate (HCOONa), 2 mM CoA, 0.1 mM coenzyme B_12_, 5 mM ATP, 5 mM NADH, 5 mM NADPH, 1 mM glycine and 0.1 mM pyridoxalphosphat. The enzymes were used at the concentrations shown in Supplementary Table 1, while the concentration of Frd was adjusted as displayed in Fig. 3a. The reactions were started with 250 µM glyoxylate, and the tubes were shaken at 450 rpm at 30 °C. Samples (8 µl) were taken at 30, 60, 90 and 120 min and quenched.

#### CETCH with module 4b (Fig. 3b and Extended Data Fig. 2a)

The assay was performed in triplicates in an assay volume of 75 µl and included 100 mM HEPES-KOH pH 7.5, 10 mM MgCl_2_, 20 mM phosphocreatine, 50 mM bicarbonate (NaHCO_3_), 20 mM formate (HCOONa), 2 mM CoA, 0.1 mM coenzyme B_12_, 5 mM ATP, 5 mM NADH, 5 mM NADPH, 1 mM glycine and 0.1 mM pyridoxalphosphat. The enzymes were used at the concentrations shown in Supplementary Table 1. Mdh was either used at 7.9 U ml^−1^ or 0.8 U ml^−1^. The reactions were started with 250 µM glyoxylate, and the tubes were shaken at 450 r.p.m. at 30 °C. Samples (8 µl) were taken at 0, 30, 60, 90, 120 and 240 min and quenched.

#### CETCH with module 4c (Fig. 3c)

The assay was performed in triplicates in an assay volume of 75 µl and included 100 mM HEPES-KOH pH 7.5, 10 mM MgCl_2_, 20 mM phosphocreatine, 50 mM bicarbonate (NaHCO_3_), 20 mM formate (HCOONa), 2 mM CoA, 0.1 mM coenzyme B_12_, 5 mM ATP, 5 mM NADH, 5 mM NADPH, 1 mM glycine and 0.1 mM pyridoxalphosphat. The enzymes were used at the concentrations shown in Supplementary Table 1, one assay with and one without Adk. The reactions were started with 250 µM glyoxylate, and the tubes were shaken at 450 rpm at 30 °C. Samples (8 µl) were taken at 0, 30, 60, 90 and 120 min (+240 min sample for the experiment with Adk) and quenched.

#### CETCH with module 4d (Extended Data Fig. 2b,c)

The assay was performed in triplicates in an assay volume of 90 µl and included 100 mM HEPES-KOH pH 7.5, 5 mM MgCl_2_, 20 mM phosphocreatine, 50 mM bicarbonate (NaHCO_3_), 20 mM formate (HCOONa), 0.25, 0.5 or 1 mM CoA, 0.1 mM coenzyme B_12_, 5 mM ATP, 5 mM NADH, 5 mM NADPH, 1 mM glycine and 0.1 mM pyridoxalphosphat. The enzymes were used at the concentrations shown in Supplementary Table 1. The reactions were started with 500 µM glyoxylate, and the tubes were shaken at 450 r.p.m. at 30 °C. Samples (12 µl) were taken at 0, 15, 30, 60, 120 and 240 min and quenched.

#### CETCH with module 4d (Fig. 3d)

The assay was performed in triplicates in an assay volume of 65 µl and included 100 mM HEPES-KOH pH 7.5, 10 mM MgCl_2_, 20 mM phosphocreatine, 50 mM bicarbonate (NaHCO_3_), 20 mM formate (HCOONa), 1 or 2.5 mM CoA, 0.1 mM coenzyme B_12_, 5 mM ATP, 5 mM NADH, 5 mM NADPH, 1 mM glycine and 0.1 mM pyridoxalphosphat. The enzymes were used at the concentrations shown in Supplementary Table 1. The reactions were started with 500 µM glyoxylate, and the tubes were shaken at 450 r.p.m. at 30 °C. Samples (8 µl) were taken at 0, 30, 60, 90, 120 and 240 min and quenched.

#### CETCH with all modules with and without DEBS (Fig. 4)

The assays were performed in duplicates in an assay volume of 150 µl and included 100 mM HEPES-KOH pH 7.5, 10 mM MgCl_2_, 20 mM phosphocreatine, 50 mM bicarbonate (NaHCO_3_), 20 mM formate (HCOONa), 2 or 1 (module 4d) mM CoA, 0.1 mM coenzyme B_12_, 5 mM ATP, 5 mM NADH, 5 mM NADPH, 1 mM glycine and 0.1 mM pyridoxalphosphat. The enzymes were used at the concentrations shown in Supplementary Table 1. For the assay with module 4a, the 4Fe–4S cluster of Acn was reconstituted before the assay. Therefore, the purified enzyme was incubated with 5 mM dithiothreitol and 15 mM ammonium Fe(II), So_4_. After 30 min on ice, the buffer was exchanged with the storage buffer using a Zeba Micro Spin Desalting Column, 7 K MWCO, 0.5 ml (Thermo Scientific) according to the provided protocol. To avoid NADPH oxidation by Frd during preparation of the assay, Frd was added as the last enzyme before starting the reaction in the assay with module 4a. The reactions were started with 250 µM glyoxylate or 125 µM propionyl-CoA (CETCH controls), and the tubes were shaken at 450 r.p.m. at 30 °C. Samples (13 µl) were taken at 0, 0.5, 1, 1.5, 2, 4, 6, 8, 10, 12 and 24 h and quenched.

#### DEBS assays (Fig. 4 and Extended Data Fig. 4)

All DEBS assay were carried out in duplicates and contained 100 mM HEPES-KOH pH 7.5, 200 mM NaCl, 4 mM NADPH (0.7 mM for spectrophotometric assays), 4 µM Epi and 2 µM of each DEBS protein (Supplementary Fig. 3). The reactions were started on the addition of 0.8 mM propionyl-CoA and 1 mM methylmalonyl-CoA (which were omitted for the negative control). For quantification, samples were taken after 0, 20, 40, 60, 80, 100, 120, 150 and 180 min, quenched with a final concentration of 5% (vol/vol) formic acid and stored at −80 °C until measurement.

Reduction rates were measured on a Cary 60 UV–vis spectrometer (Agilent) using 10 mm quartz cuvettes (Hellma) following NADPH absorption at 360 nm (Δ*ε*_360_ = 3.4 mM^−1^ cm^−1^). 6-DEB quantification was performed following established methods^25^, which use the NADPH oxidation rate in stoichiometric equivalence to product formation (6 mol NADPH per 1 mol 6-DEB). For this, we spectrophotometrically measured the incremental reduction rates of a DEBS control assay and correlated those to the corresponding 6-DEB EICs from an identical time course of LC–MS measurements to make a standard curve (Extended Data Fig. 4). The latter was then used for 6-DEB quantification of the setups containing feedback modules.

### Steady-state kinetics of Bbd (Supplementary Fig. 2a)

The activity of Bbd was measured in a coupled assay using Etr1p. The assay was done in an volume of 150 µl in a high precision quartz cuvette (10 mm, Hellma Analytics) at 30 °C and contained 200 mM HEPES-KOH pH 7.5, 0.5 mM NADPH, 0.009 µg Bbd and 3 µg Etr1p. The reactions were started with 5, 10, 25, 50, 100, 200 and 300 µM (*R*)-3-hydroxybutyryl-CoA. The consumption of NADPH was observed at 365 nm (∆*ε*_365_ = 3.3 mM^−1^ cm^−1^) on a Cary 60 UV–vis spectrophotometer using the Cary WinUV Kinetics Application (Agilent Technologies). The Michaelis−Menten constants were calculated using Graphpad Prism.

### Steady-state kinetics of Mtk (Supplementary Fig. 2b)

Mtk (comprising subunits MtkA and MtkB; MtkAB) activity was determined by coupling the reaction to Mcl, producing acetyl-CoA and glyoxylate. The latter was further reduced to glycolate using a corresponding reductase (Gox) and monitored following NADPH consumption at 360 nm (∆*ε*_360_ = 3.4 mM^−1^ cm^−1^) on a Cary 60 UV–vis spectrometer using the Cary WinUV Kinetics Application (Agilent) and high precision quartz cuvettes (10 mm, Hellma Analytics). The assay contained 200 mM HEPES-KOH pH 7.5, 100 mM MgCl_2_, 5 mM ATP, 2 mM CoA and 0.8 mM NADPH, 14.73 µg MtkAB (equivalent amounts of both subunits), 3 µg Mcl, 2.1 µg Gox and varying amounts of l-malate in a final volume of 100 µl. The Michaelis–Menten constants were calculated using Graphpad Prism.

### LC–MS measurements

#### Analysis of CoA esters

All CoA esters were measured on a triple quadrupole mass spectrometer (Agilent Technologies 6495 Triple Quad LS–MS) equipped with UHPLC (Agilent Technologies 1290 Infinity II) using a 50 × 2.1 mm C18 column (Kinetex 1.7 µm EVO C18 100 Å) at 25 °C. The injection volume was 2 µl of the diluted samples (1:10 in water). The flow was set to 0.250 ml min^-1^, and the separation was performed using 50 mM ammonium formate pH 8.1 (buffer A) and acetonitrile (buffer B). We quantified the CoA esters using external standard curves prepared in the 1:10 diluted (water) sample matrix. The gradient for LC–MS analysis and the parameters for the multiple reaction monitoring (MRMs) are displayed in Supplementary Tables 4 and 5, respectively. Data analysis was done using Agilent MassHunter Workstation Software.

#### Glycolate quantification

Glycolate was measured on a triple quadrupole mass spectrometer (Agilent Technologies 6495 Triple Quad LS–MS) equipped with UHPLC (Agilent Technologies 1290 Infinity II) using a 150 × 2.1 mm C18 column (Kinetex 1.7 µm EVO C18 100 Å) at 25 °C. The injection volume was 1 µl. The diluted samples (1:10 in water) as well as the external standard curve were diluted 1:2 with 10 µM ^13^C-labeled glycolate as the internal standard. The flow was set to 0.100 ml min^−1^, and the separation was performed using dH_2_O with 0.1% formic acid (buffer A) and methanol with 0.1% formic acid (buffer B). The gradient for LC–MS analysis and the parameters for the MRMs are displayed in Supplementary Tables 6 and 7, respectively. Data analysis was done using Agilent MassHunter Workstation Software.

#### Malate quantification

Malate was measured on a triple-quadrupole mass spectrometer (Agilent Technologies 6495 Triple Quad LS–MS) equipped with UHPLC (Agilent Technologies 1290 Infinity II) using a 150 × 2.1 mm C18 column (Kinetex 1.7 µm EVO C18 100 Å) at 25 °C. The injection volume was 5 µl. The diluted samples (1:10 in water) as well as the external standard curve were diluted 1:2 with 10 µM ^13^C-labeled malate as the internal standard. The flow was set to 0.150 ml min^−1^, and the separation was performed using dH_2_O with 0.1% formic acid (buffer A) and methanol with 0.1% formic acid (buffer B). The gradient for LC–MS analysis and the parameters for the MRMs are displayed in Supplementary Tables 8 and 9, respectively. Data analysis was done using Agilent MassHunter Workstation Software.

#### HPLC–MS analysis of 6-DEB

In total, 5 µl of the quenched assays were analyzed via HPLC-ESI-TOF on a 6550 iFunnel Q-TOF LC–MS (Agilent) with a 1.8-µm Zorbax SB-C18 column, 50 × 2.1 mm (Agilent) and using H_2_O (buffer A) and acetonitrile (buffer B) both containing 0.1% formic acid. The gradient condition were as follows: 0 min 5% B, 1 min 5% B, 6 min 95% B, 6.5 min 95% B and 7 min 5 % B with a flow rate of 250 µl min^−1^. Capillary voltage was set at 3.5 kV, and nitrogen gas was used as nebulizing (20 psig), drying (13 l min^-1^, 225 °C) and sheath (12 l min^−1^, 400 °C) gas. MS data were acquired with a scan range of 50–1,200 *m*/*z*. Data were analyzed using MassHunter Analysis software (Agilent). Evaluated 6-DEB (room temperature 3.26 min) adducts are shown in Supplementary Table 10.

#### CoA standards synthesis

All CoA thioesters were synthesized and purified according to previously established protocols (refs. ^52,53^).

### Reporting summary

Further information on research design is available in the Nature Research Reporting Summary linked to this article.

## Online content

Any methods, additional references, Nature Research reporting summaries, source data, extended data, supplementary information, acknowledgements, peer review information; details of author contributions and competing interests and statements of data and code availability are available at 10.1038/s41589-022-01179-0.

## Supplementary information

Supplementary Information Supplementary Tables 1–10 and Supplementary Figs. 1–4.

Reporting Summary

Supplementary Data 1 Supporting data for Supplementary Fig. 1 Theoretically calculated free energy values.

Supplementary Data 2 Supporting data for Supplementary Fig. 2. Enzyme activity data.

Supplementary Data 3 Supporting data for Supplementary Fig. 4. HPLC–MS counts.

## Data Availability

All data generated or analyzed during this study are included in this published article, its Supplementary Information files. Source data are provided with this paper.

## Source data

Source Data Fig. 2 HPLC–MS counts.
Source Data Fig. 3 HPLC–MS counts.
Source Data Fig. 4 HPLC–MS counts.
Source Data Extended Data Fig. 2 HPLC–MS counts.
Source Data Extended Data Fig. 3 HPLC–MS counts.
Source Data Extended Data Fig. 4 HPLC–MS counts.
Source Data Extended Data Fig. 5 HPLC–MS counts.
Source Data Extended Data Fig. 6 HPLC–MS counts.
Source Data Extended Data Fig. 7 HPLC–MS counts.
