# Supplementary material for: Synthetic anaplerotic modules for the direct synthesis of complex molecules from CO2
Source: Nat Chem Biol. 2022 Dec 5;19(2):168–75. doi: 10.1038/s41589-022-01179-0 (PMC9889269; doi:10.1038/s41589-022-01179-0)
Supplement: Supplementary file 1 — Supplementary Tables 1–10 and Supplementary Figs. 1–4. [file 41589_2022_1179_MOESM1_ESM.pdf]

nature chemical biology

Article https://doi.org/10.1038/s41589-022-01179-0

Synthetic anaplerotic modules for the direct synthesis of complex molecules from CO_2

In the format provided by the authors and unedited

Supplementary Information

Synthetic anaplerotic modules for direct synthesis of complex molecules from CO_2

Christoph Diehl[1*], Patrick D. Gerlinger[1*], Nicole Paczia[2], Tobias J. Erb[1,3#]

[1] Department of Biochemistry & Synthetic Metabolism, Max Planck Institute for Terrestrial Microbiology, Karl-von-Frisch Str. 10, 35043 Marburg, Germany

[2] Core Facility for Metabolomics and Small Molecule Mass Spectrometry, Max Planck Institute for Terrestrial Microbiology, Karl-von-Frisch Str. 10, 35043, Marburg, Germany

[3] SYNMIKRO Center for Synthetic Microbiology, Karl-von-Frisch Str., 14, 35032 Marburg, Germany

* Equal contribution

[#] To whom correspondence should be addressed: toerb@mpi-marburg.mpg.de

Supplementary Tables 1-10

Supplementary Figures 1-4

Supplementary Table 1. Concentrations of enzymes used in the assays. Setups for modules 4a-d additionally contained all required enzymes from modules 1, 2 and 3. CA – carbonic anhydrase; Ppk – polyphosphate kinase, Cpk – creatinphosphate kinase. *Wilbur-Anderson unit. **Cell extract. All commercially available enzymes were purchased from Sigma-Aldrich.

	**Module 1**		**Module 4a**		**Module 4b**		**Module 4c**		**Module 4d**				
**Enzyme**	U/ml	µM	U/ml	µM	U/ml	µM	U/ml	µM	U/ml	µM		U/mg	Source
Pco	1	3.00	1	3.00	1	3.00	1	3.00	1	3.00		12	[1]
Ccr	1	0.58	1	0.58	1	0.58	1	0.58	1	0.58		110	[1]
Epi	2.5	0.60	2.5	0.60	2.5	0.60	2.5	0.60	2.5	0.60		440	[12]
Mcm	0.2	0.36	0.2	0.36	0.2	0.36	0.2	0.36	0.2	0.36		20	[13]
Scr	4	2.68	4	2.68	4	2.68	4	2.68	4	2.68		29	[14]
Ssr	0.1	0.76	0.1	0.76	0.1	0.76	0.1	0.76	0.1	0.76		4	[1]
Hbs	0.2	5.12	0.2	5.12	0.2	5.12	0.2	5.12	0.2	5.12		2	[15]
Gbd	0.2	0.56	0.2	0.56	0.2	0.56	0.2	0.56	0.2	0.56		26	[15]
Ecm	0.2	0.55	0.2	0.55	0.2	0.55	0.2	0.55	0.2	0.55		7	[12]
Mco	0.1	21.4	0.1	21.4	0.1	21.4	0.1	21.4	0.1	21.4		0.1	[1]
Mch	5	1.26	5	1.26	5	1.26	5	1.26	5	1.26		1500	[16]
Mcl	0.5	13.6	0.5	13.6	0.5	13.6	0.5	13.6	0.5	13.6		5	[17]
Cat	1.5	1.37	1.5	1.37	1.5	1.37	1.5	1.37	1.5	1.37		11740	[18]
Fdh	0.5	14.4	0.5	14.4	0.5	14.4	0.5	14.4	0.5	14.4		1	[19]
Cpk	4	0.39	4	0.39	4	0.39	4	0.39	4	0.39		150	commercial
Ppk	0.5	2										12	[20]
CA	*	0.02	*	0.02	*	0.02	*	0.02	*	0.02		*2000	commercial
Gor	0.1	1.10										-	[21]
Bha			0.5	2.26	0.5	2.26	0.5	2.26	0.5	2.26		116	[22]
Bhd			0.5	1.37	0.5	1.37	0.5	1.37	0.5	1.37		92	[22]
Isr			5	14.8	5	14.8	5	14.8	5	14.8		358	[22]
Agt			1.2	1.93	1.2	1.93	1.2	1.93	1.2	1.93		77	[22]
Mdh			7.91	1.33	0.79	0.13	7.91	1.33	7.91	1.33		1611	[23]
Mtk					0.5		0.5		0.5			1.5	This Work
Fum			0.5	0.66								340	[24]
Frd			0.01	1.05								0.007	[5]
Scs			0.5	1.00	0.5	1.00						19	[25]
Cit					0.5	8.98						4	[26]
Acn					0.5	15.88						6	[27]
Icl					5	12.54						38	[28]
Pcc*							0.1	7.69				1	[29]
Mcr							0.5	0.28				10	[7]
Pcs							0.1	3.09				1	[30]
Adk							15	0.18				1247	[31]
Aat									0.5	17.74		5	[32]
Aar									0.5	28.27		**0.6	[33]
Bbd									0.5	52.47		965	This work
DEBS		(2) each protein		(2) each protein		(2) each protein		(2) each protein		(2) each protein			-

Supplementary Table 2. Yield comparison of the different modules. mm-CoA: methylmalonyl-CoA. Carbons/mm-CoA and Total carbons in product are calculated for the methylmalonyl backbone (C4). Note that all setups include modules 1-3. * calculations include the carbon lost through decarboxylative condensation.

	Substrate (glyoxylate) [µM]	Initial carbon concentration [µM]		Product mm-CoA [µM]	Carbon per mm-CoA	Total carbon concentration from product [µM]	Yield [%]
4a	250	500		130	4	520	104
4b	250	500		110	4	441	88
4c	250	500		498	4	1992	398
4d	250	500		69	4	276	55
				Product 6-dEB [µM]	Carbons per 6-dEB*		
4a+5	250	500		6	27	163	42
4b+5	250	500		2.8	27	76	15
4c+5	250	500		31.9	27	863	173
4d+5	250	500		0	27	0	0

Supplementary Table 3. List of all pathway enzymes used in this work. Note that auxiliary enzymes are listed in Table S1.

#	Name	Full name	Catalyzed reaction	Origin	Comment	Source
1	Ccr	crotonyl-CoA carboxylase/reductase	Crotonyl-CoA + NADPH + CO2 ⇌ Ethylmalonyl-CoA + NADP+	*M. extorquens*		1
2	Epi	methylmalonyl-/ethylmalonyl-CoA epimerase	(2S)-Ethylmalonyl-CoA ⇌ (2R)-Ethylmalonyl-CoA	*R. sphaeroides*		1
	Ecm	ethylmalonyl-CoA mutase	(2R)-Ethylmalonyl-CoA ⇌ Methylsuccinyl-CoA	*R. sphaeroides*		1
3	Mco	methylsuccinyl-CoA oxidase	Methylsuccinyl-CoA + O2 ⇌ Mesaconyl-CoA + H2O2	*R. sphaeroides*		1
4	Mch	mesacony-CoA hydratase	Mesaconyl-CoA + H2O(l) ⇌ beta-Methylmalyl-CoA	*R. sphaeroides*		1
5	Mcl	β-methylmalyl-CoA lyase	beta-Methylmalyl-CoA ⇌ Glyoxylate + Propionyl-CoA	*R. sphaeroides*		1
6	Pco	propionyl-CoA oxidase	Propionyl-CoA + O2 ⇌ Acrylyl-CoA + H2O2	*A. thaliana*	A. thaliana short chain acyl-CoA oxidase 4 T134L	1
7	Ccr	Crotonyl-CoA carboxylase/reductase	Acrylyl-CoA + NADPH + CO2 ⇌ Methylmalonyl-CoA + NADP+	*M. extorquens*		1
8	Epi	methylmalonyl-/ethylmalonyl-CoA epimerase	(2S)-Ethylmalonyl-CoA ⇌ (2R)-Ethylmalonyl-CoA	*R. sphaeroides*		1
	Mcm	methylmalonyl-CoA mutase	Methylmalonyl-CoA ⇌ Succinyl-CoA	*R. sphaeroides*		1
9	Scr	succinyl-CoA reductase	Succinyl-CoA + NADPH ⇌ Succinic semialdehyde + NADP+ + CoA	*C. kluyveri*		1
10	Ssr	succinic semialdehyde reductase	Succinic semialdehyde + NADPH ⇌ 4-Hydroxybutyric acid + NADP+	*H. sapiens*		1
11	Hbs	4-hydroxybutyryl-CoA synthetase	4-Hydroxybutyric acid + ATP + CoA ⇌ 4-Hydroxybutyryl-CoA + ADP + Pi	*N. maritimus*		1
12	Hbd	4-hydroxybutyryl-CoA dehydratase	4-Hydroxybutyryl-CoA ⇌ Crotonyl-CoA + H2O(l)	*N. maritimus*		1
13	Bha	β-hydroxyaspartate aldolase	3-hydroxyaspartate ⇌ Iminosuccinate + H2O	*P. denitrificans*	bhcC	2
14	Bhd	β-hydroxyaspartate dehydratase	Glyoxylate + Glycine ⇌ 3-hydroxyaspartate	*P. denitrificans*	bhcB	2
15	Isr	imminosuccinate reductase	Iminosuccinate + NADPH ⇌ Aspartate + NADP+	*P. denitrificans*	bhcD	2
16	Agt	aspartate-glyoxylate aminotransferase	Aspartate + Glyoxylate ⇌ Oxaloacetate + Glycine	*P. denitrificans*	bhcA	2
17	Mdh	malate dehydrogenase	Oxaloacetate + NADH ⇌ Malate + NAD+	*E. coli*		3
18	Mtk	malyl-CoA synthetase	Malate + ATP + CoA ⇌ Malyl-CoA + ADP + Pi	*M. extorquens*	mtkAB; subunit beta A, subunit alpha B	3
19	Mcl	β-methylmalyl-CoA lyase	Malyl-CoA ⇌ Glyoxylate + Acetyl-CoA	*R. sphaeroides*		1

Supplementary Table 3 continued

20	Fum	fumarate hydratase	Malate ⇌ Fumarate + H2O	*E. coli*		4
21	Frd	fumarate reductase	Fumarate + NADH ⇌ Succinate + NAD+	*T. brucei*		5
22	Scs	succinyl-CoA synthetase	Succinate + ATP + CoA ⇌ Succinyl-CoA + ADP + Pi	*E. coli*	sucC subunit beta, sucD subunit alpha	4
23	Cit	citrate synthase	Acetyl-CoA + Oxaloacetate + H2O(l) ⇌ Citrate + CoA	*Synechocystis sp.6803*		This work
24	Acn	aconitate hydratase A	Citrate ⇌ Isocitrate	*E. coli*		4
25	Icl	isocitrate lyase	Isocitrate ⇌ Glyoxylate + Succinate	*E. coli*		4
26	Pcc*	propionyl-CoA carboxylase	Acetyl-CoA + HCO3- + ATP ⇌ Malonyl-CoA + AMP + PPi	*M. extorquens*	M. extorquens propionyl-CoA carboxylase D407I	6
27	Mcr	malonyl-CoA reductase	Malonyl-CoA + NADPH ⇌ Malonate semialdehyde + NADP+ + CoA	*C.aurantiacus*		7
28	Mcr	malonyl-CoA reductase	Malonate semialdehyde + NADPH ⇌ 3-Hydroxypropionate + NADP+	*C.aurantiacus*		7
29	Pcs	propionyl-CoA synthase	3-Hydroxypropionate + ATP + CoA ⇌ 3-Hydroxypropionyl-CoA + AMP + PPi	*Erythrobacter NAP1*		8
30	Pcs	propionyl-CoA synthase	3-Hydroxypropionyl-CoA ⇌ Acrylyl-CoA + H2O(l)	*Erythrobacter NAP1*		8
31	Pcs	propionyl-CoA synthase	Acrylyl-CoA + NADPH ⇌ Propionyl-CoA + NADP+	*Erythrobacter NAP1*		8
32	pha	acetoacetyl-CoA thiolase	2 Acetyl-CoA ⇌ Acetoacetyl-CoA + CoA	*C.necator*		9
33	phb	acetoacetyl-CoA reductase	Acetoacetyl-CoA + NADPH ⇌ (S)-3-Hydroxybutyryl-CoA + NADP+	*C.necator*		9
34	phj	enoyl-CoA hydratase	(S)-3-Hydroxybutyryl-CoA ⇌ Crotonyl-CoA + H2O(l)	*P.aeruginosa*		10
35	DEBS	6-deoxyerythronolide B synthase	Propionyl-CoA + 6 NADPH + 6 Methylmalonyl-CoA ⇌ 6-Deoxyerythronolide B + 6 CO2 + 7 CoA + 6 NADP+ + H2O	*S. erythrea*	summarized reaction sequence	11

Supplementary Table 1. LC-MS gradient for the separation of CoA thioesters.

Time [min]	A [%]	B [%]
0	100	0
2	100	0
5	94	6
8	77	23
10	20	80
11	20	80
12	100	0
12.5	100	0

Supplementary Table 2. MRM transitions for the quantification of CoA thioesters.

Compound	Precursor Ion	Product Ion	Dwell	Fragmentor	Collision Energy	Cell Accelerator Volt.	Polarity
Malyl-CoA (Quantifier)	884.1	377.1	25	380	37	5	Positive
Malyl-CoA (Qualifier)	884.1	428	25	380	29	5	Positive
Acetyl-CoA (Quantifier)	810.1	302.2	25	380	35	5	Positive
Acetyl-CoA (Qualifier)	810.1	428	25	380	35	5	Positive
Ethylmalonyl-CoA (Quantifier)	882.1	331.2	25	380	41	5	Positive
Ethylmalonyl-CoA (Qualifier)	882.1	428	25	380	29	5	Positive
Methylsuccinyl-CoA (Quantifier)	882	375.1	25	380	33	5	Positive
Methylsuccinyl-CoA (Qualifier)	882	428	25	380	29	5	Positive
Mesaconyl-CoA (Quantifier)	880.1	375.1	25	380	25	5	Positive
Mesaconyl-CoA (Qualifier)	880.1	428	25	380	35	5	Positive
Succinyl-CoA (Quantifier)	868.1	361.1	25	380	35	5	Positive
Succinyl-CoA (Qualifier)	868.1	428.1	25	380	35	5	Positive
Methylmalonyl-CoA (Quantifier)	868.1	317.1	25	380	41	5	Positive
Methylmalonyl-CoA (Qualifier)	868.1	428	25	380	31	5	Positive
Malonyl-CoA (Quantifier)	854.1	245	25	380	32	5	Positive
Malonyl-CoA (Qualifier)	854.1	428	25	380	28	5	Positive
γ-hydroxybutyryl-CoA (Quantifier)	854.1	347.1	25	380	37	5	Positive
γ-hydroxybutyryl-CoA (Qualifier)	854.1	428	25	380	30	5	Positive
Crotonyl-CoA (Quantifier)	836.1	329	25	380	33	5	Positive
Crotonyl-CoA (Qualifier)	836.1	428	25	380	26	5	Positive
Propionyl-CoA (Quantifier)	824.1	317.1	25	380	31	5	Positive
Propionyl-CoA (Qualifier)	824.1	428	25	380	28	5	Positive

Supplementary Table 5 continued.

Methylsuccinyl-CoA (Quantifier)	824.1	317.1	25	380	31	5	Positive
Methylsuccinyl-CoA (Qualifier)	824.1	428	25	380	28	5	Positive
B-methylmalyl-CoA (Quantifier)	898.1	391.1	25	380	39	5	Positive
B-methylmalyl-CoA (Qualifier)	898.1	428.1	25	380	33	5	Positive

Supplementary Table 3. LC-MS gradient for the analysis of glycolate.

Time [min]	A [%]	B [%]
0	100	0
4	100	0
6	0	100
7	0	100
7.1	100	0
12	100	0

Supplementary Table 4. MRM transitions for the quantification of glycolate.

Compound	Precursor Ion	Product Ion	Dwell	Fragmentor	Collision Energy	Cell Accelerator Volt.	Polarity
^{12}C-Glycolate (Quantifier)	75	47	150	380	9	5	Negative
^{12}C-Glycolate (Qualifier)	75	75	150	380	0	5	Negative
^{13}C-Glycolate (Quantifier)	77	48	150	380	9	5	Negative
^{13}C-Glycolate (Qualifier)	77	77	150	380	0	5	Negative

Supplementary Table 5. LC-MS gradient for the analysis of malate.

Time [min]	A [%]	B [%]
0.0	85	15
7.0	0	100
9.0	0	100
9.1	85	15
15.0	85	15

Supplementary Table 6. MRM transitions for the quantification of malate.

Compound	Precursor Ion	Product Ion	Dwell	Fragmentor	Collision Energy	Cell Accelerator Volt.	Polarity
^{12}C-Malate (Quantifier)	133	115	150	80	11	5	Negative
^{12}C-Malate (Qualifier)	133	133	150	80	0	5	Negative
^{13}C-Malate (Quantifier)	137	119	150	80	11	5	Negative

Supplementary Table 7. Analyzed 6-dEB adducts.

**Adduct**	[M+H]+	[M+Na]+	[M-H_2O+H]+
**m/z**	387.274116	409.256058	369.263551

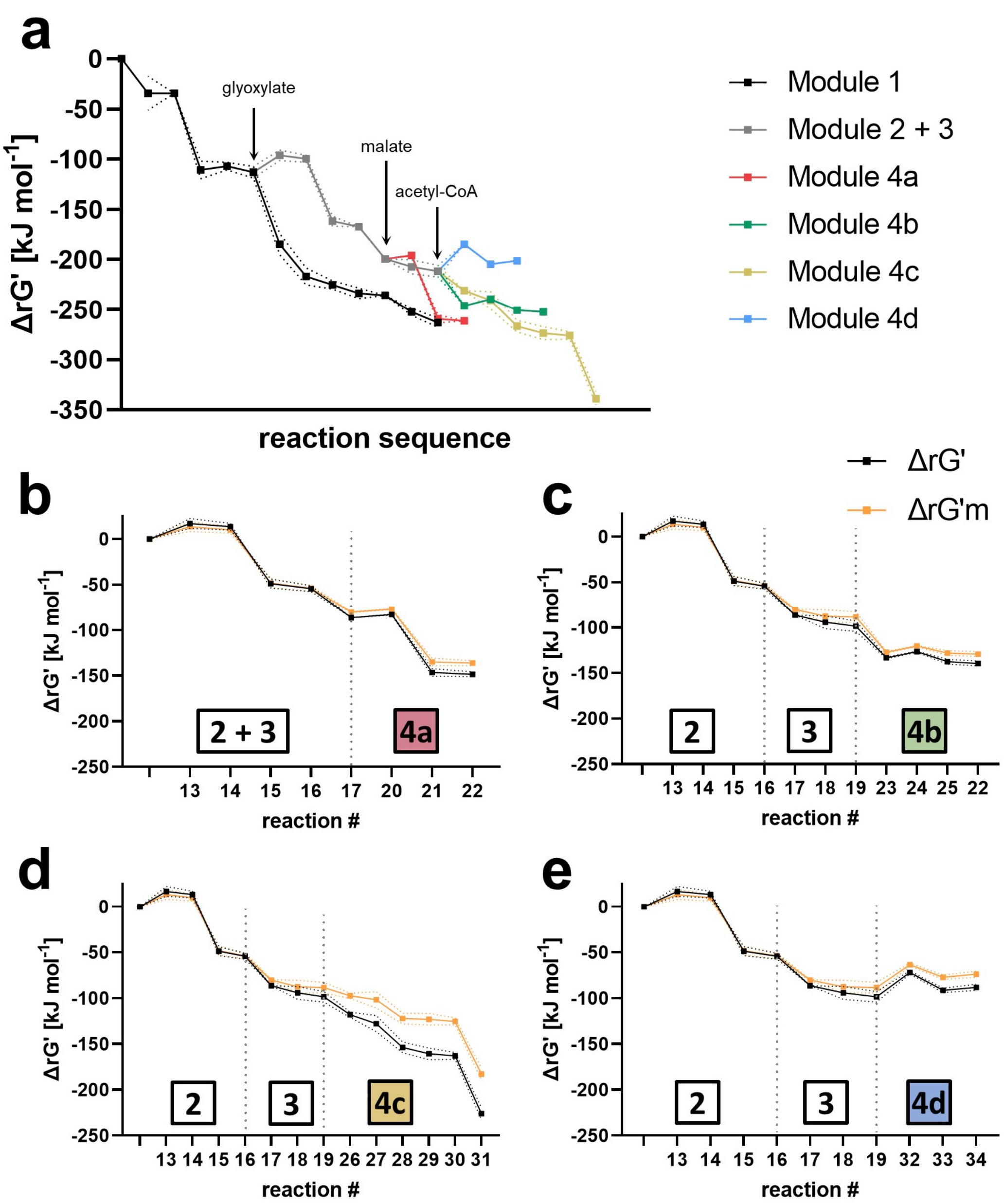

Supplementary Fig. 1. Analysis of the thermodynamic changes of all reaction modules as Gibbs free energy profile. Shown are the free energy profiles of modules and module combinations to the respective feedback intermediate. **a** Overview of consecutive reactions, starting with the carboxylation of crotonyl-CoA to ethylmalonyl-CoA (#1). Arrows indicate intermediates that mark branching points into other modules (labeled by color). **b** Modules 2-3-4a, starting from glyoxylate and yielding succinyl-CoA. **c** Modules 2-3-4b, starting from glyoxylate and yielding succinyl-CoA, **d** Modules 2-3-4c, starting from glyoxylate and yielding propionyl-CoA, **e** Module 2-3-4d, starting from glyoxylate and yielding crotonyl-CoA. Grey dashed lines indicate transitions between modules. ΔrG' (custom reactant concentrations, see below) and ΔrG'm (default concentration of 1 mM for all reactants) values were estimated using the eQuilibrator v3.0 tool[34] at pH 7.5, I = 0.25 and pMg = 3. All substrate and product (CoA, acids, aldehydes) concentrations were set to 250 µM, with the following exceptions: #23 200 µM acetyl-CoA and 50 µM oxaloacetate, #24-25 all reactants 50 µM (and 250 µM glyoxylate), #32-34 125 mM acetoacetyl-CoA and every following reactant. Concentrations of other metabolites were set as follows: NAD(P)H = 4.5 mM; NAD(P)+ = 0.5 mM; ATP = 3 mM; ADP = 1.4 mM; AMP = 0.5 mM; CoA = 1 mM; CO_2(g) = 3.31 mM; CO_2(total) = 50 mM.

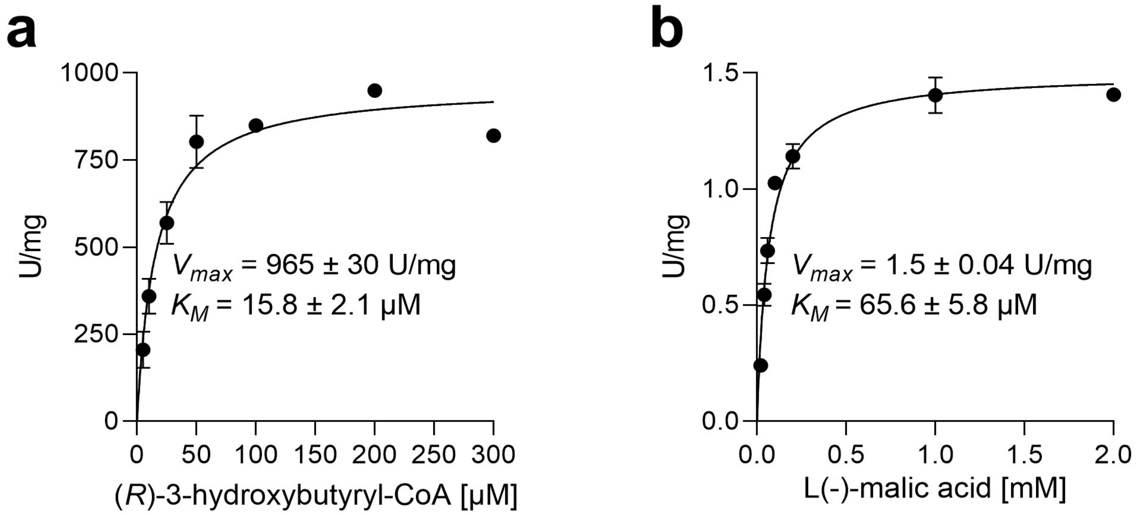

Supplementary Fig. 2. Michaelis-Menten Plots and kinetic parameters for Bbd (a) and Mtk (b). All displayed data points were measured in technical triplicates and are displayed as mean ± SD.

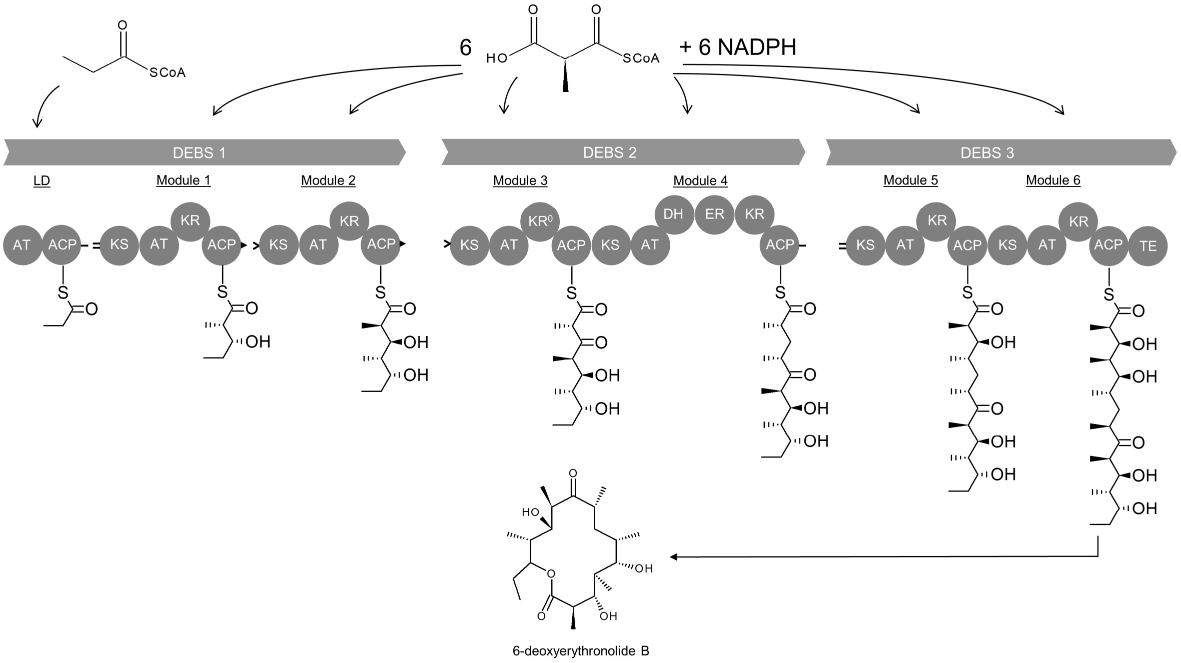

Supplementary Fig. 3. 6-deoxyerythronolide B synthase (DEBS). Displayed are the genetic architecture (DEBS 1-3), as well as the *in vitro* assembly line used in this assay. DEBS 1 was dissected into lone standing loading domain (LD), module 1 and module 2 which were connected through linker domains, as described before[11]. Each molecule 6-dEB is derived from one molecule propionyl-CoA and six molecules (2*S*)-methylmalonyl-CoA, under the consumption of six reducing equivalents NADPH.

a

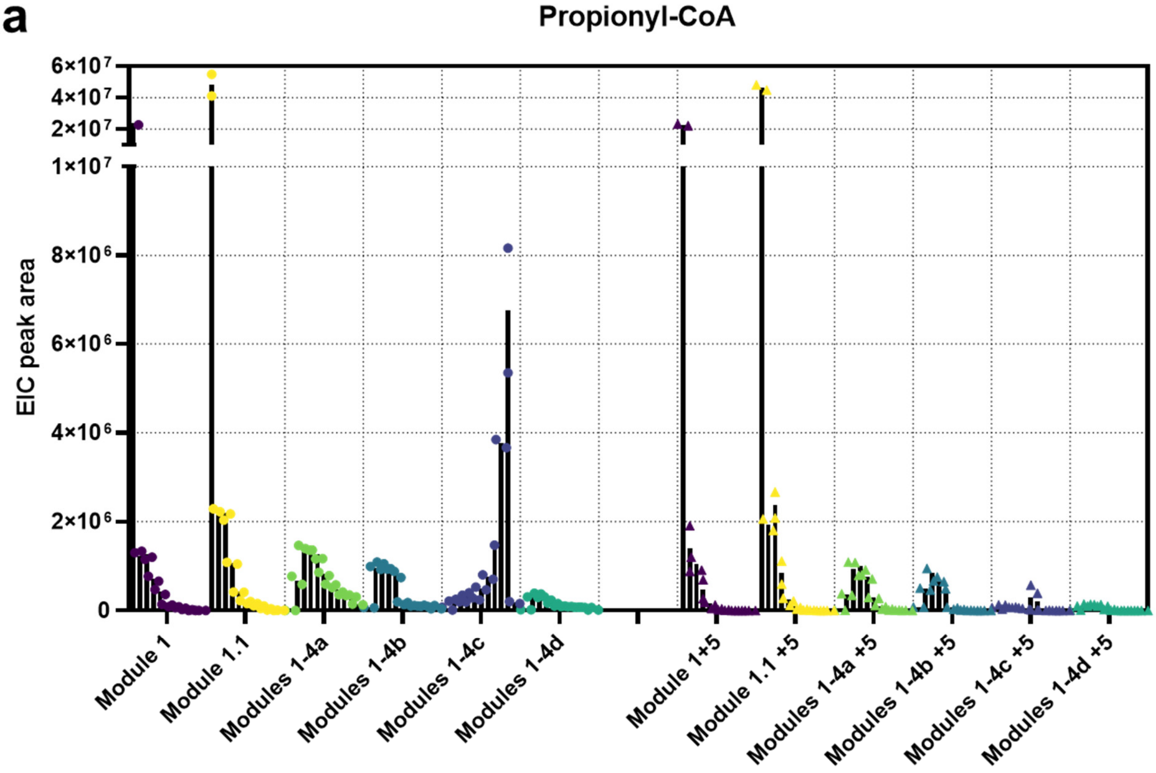
Propionyl-CoA
EIC peak area
6×10⁷
4×10⁷
2×10⁷
1×10⁷
8×10⁶
6×10⁶
4×10⁶
2×10⁶
0
Module 1
Module 1.1
Modules 1-4a
Modules 1-4b
Modules 1-4c
Modules 1-4d
Module 1+5
Module 1.1 +5
Modules 1-4a +5
Modules 1-4b +5
Modules 1-4c +5
Modules 1-4d +5

b

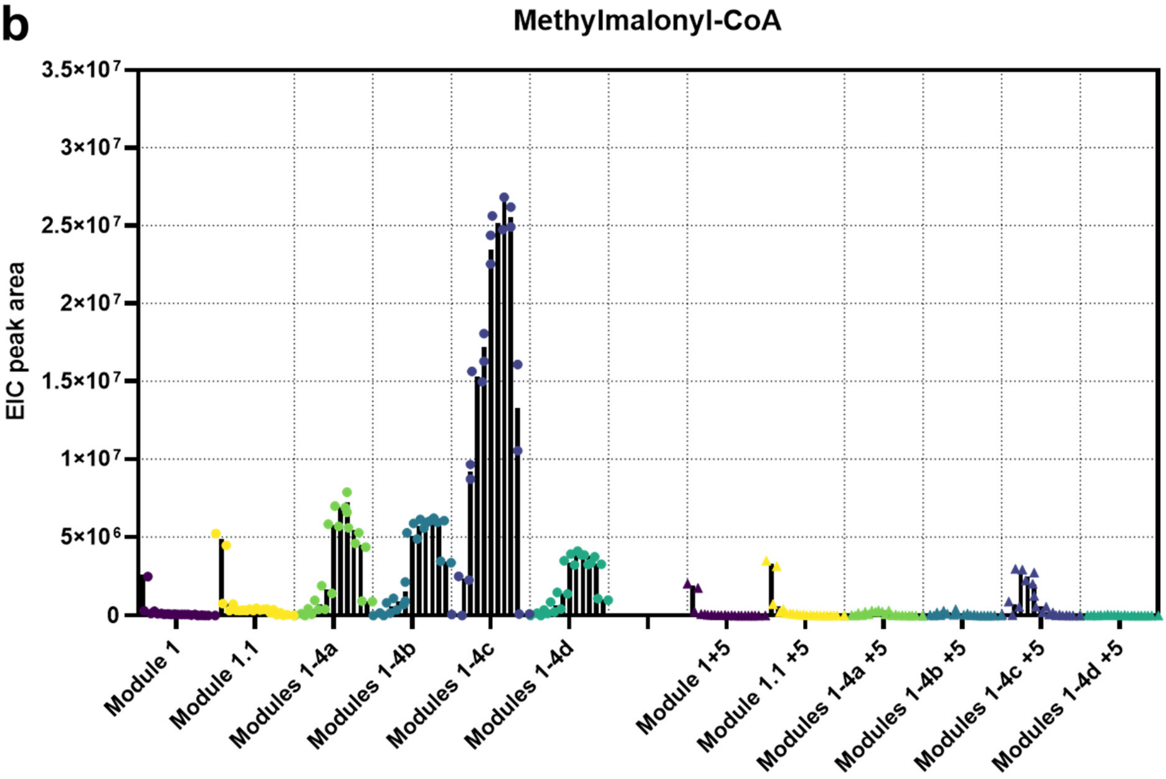
Methylmalonyl-CoA
EIC peak area
3.5×10⁷
3×10⁷
2.5×10⁷
2×10⁷
1.5×10⁷
1×10⁷
5×10⁶
0
Module 1
Module 1.1
Modules 1-4a
Modules 1-4b
Modules 1-4c
Modules 1-4d
Module 1+5
Module 1.1 +5
Modules 1-4a +5
Modules 1-4b +5
Modules 1-4c +5
Modules 1-4d +5

c

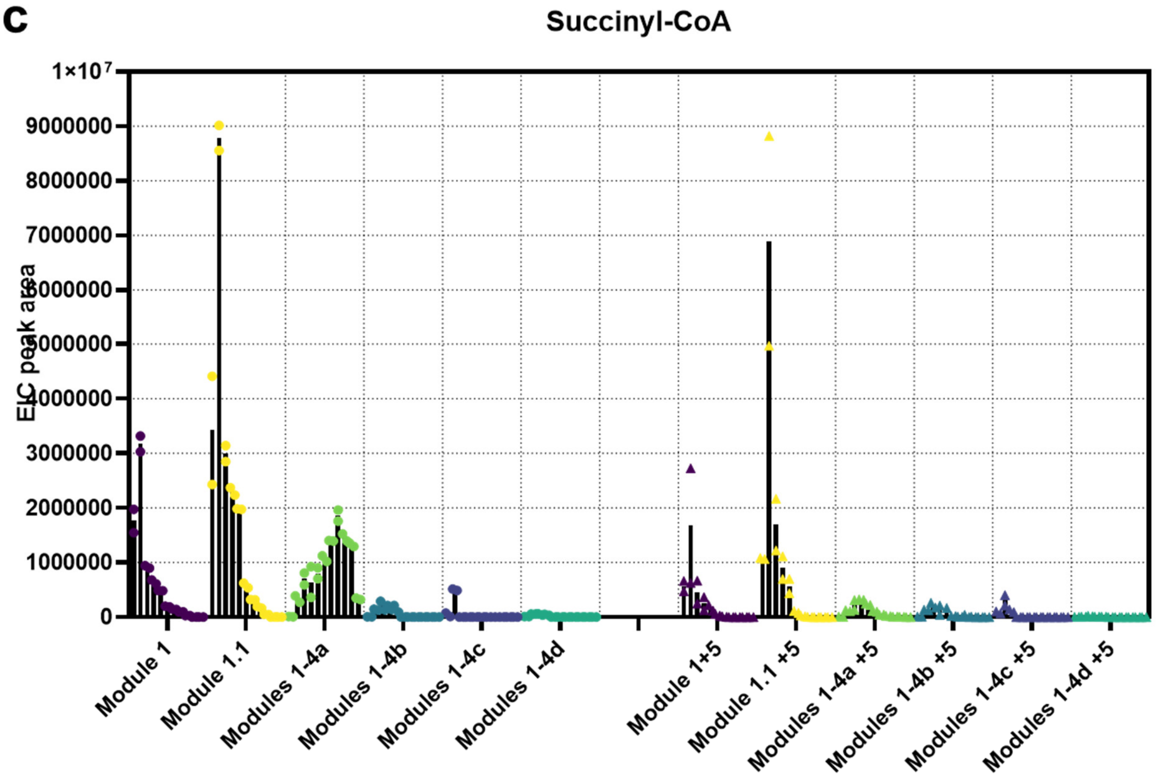
Succinyl-CoA
EIC peak area
1×10^7
9000000
8000000
7000000
6000000
5000000
4000000
3000000
2000000
1000000
0
Module 1
Module 1.1
Modules 1-4a
Modules 1-4b
Modules 1-4c
Modules 1-4d
Module 1+5
Module 1.1 +5
Modules 1-4a +5
Modules 1-4b +5
Modules 1-4c +5
Modules 1-4d +5

d

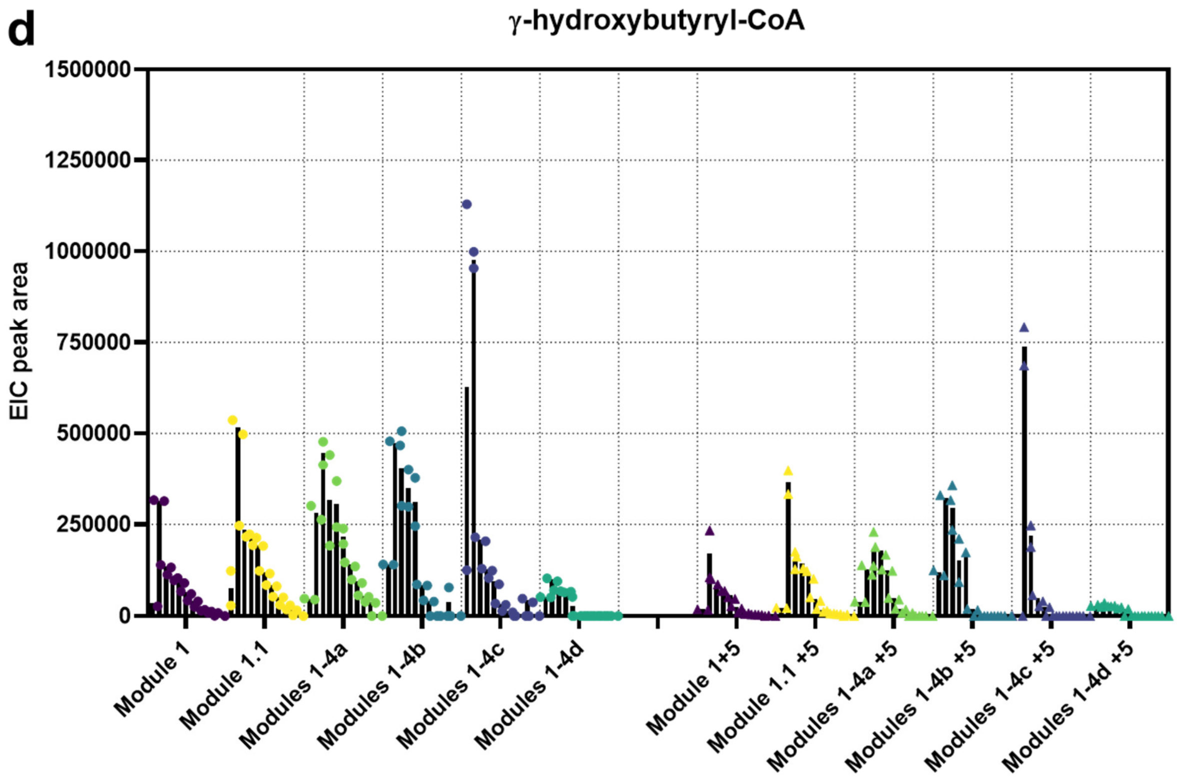
γ-hydroxybutyryl-CoA
EIC peak area
1500000
1250000
1000000
750000
500000
250000
0
Module 1
Module 1.1
Modules 1-4a
Modules 1-4b
Modules 1-4c
Modules 1-4d
Module 1+5
Module 1.1 +5
Modules 1-4a +5
Modules 1-4b +5
Modules 1-4c +5
Modules 1-4d +5

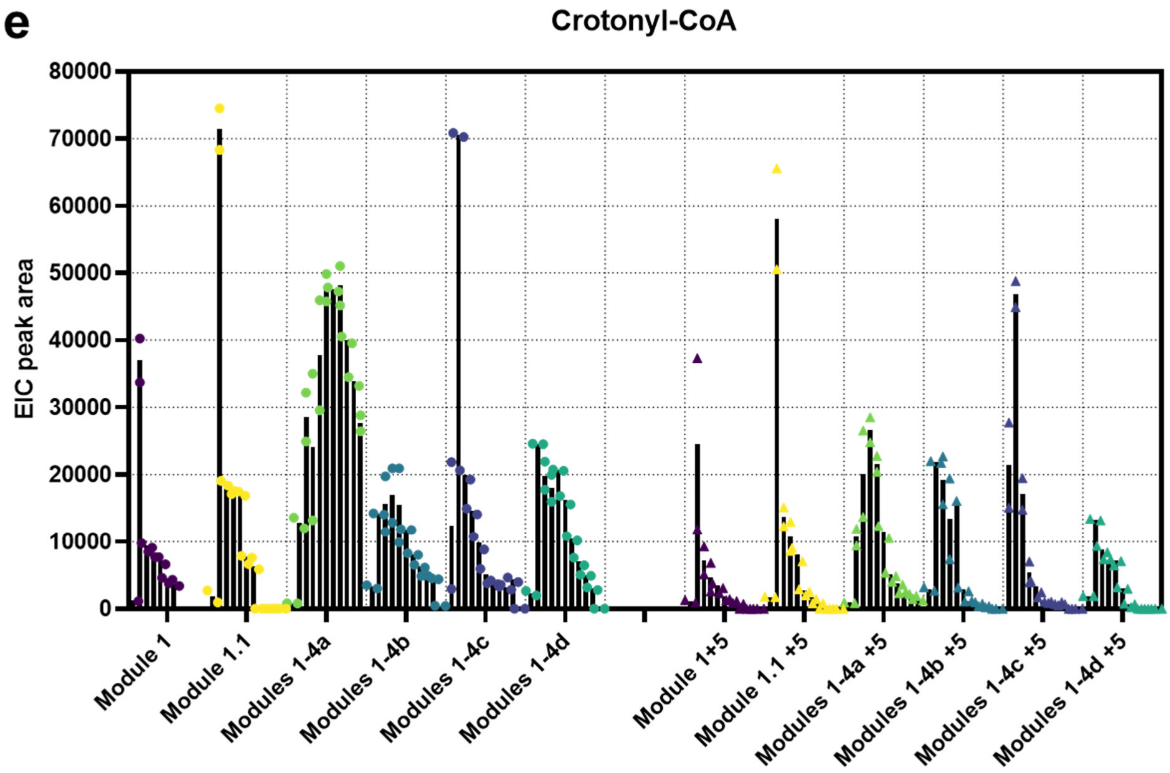
e
Crotonyl-CoA
EIC peak area
80000
70000
60000
50000
40000
30000
20000
10000
0
Module 1
Module 1.1
Modules 1-4a
Modules 1-4b
Modules 1-4c
Modules 1-4d
Module 1+5
Module 1.1 +5
Modules 1-4a +5
Modules 1-4b +5
Modules 1-4c +5
Modules 1-4d +5

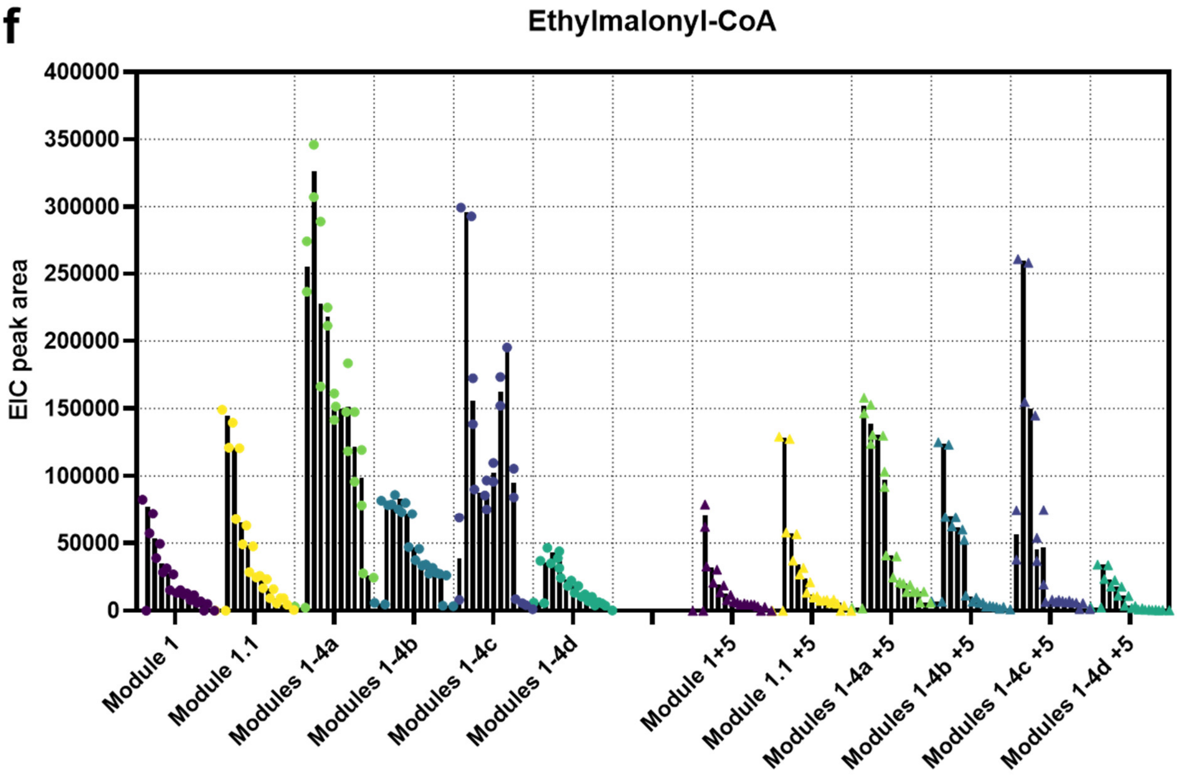
f
Ethylmalonyl-CoA
EIC peak area
400000
350000
300000
250000
200000
150000
100000
50000
0
Module 1
Module 1.1
Modules 1-4a
Modules 1-4b
Modules 1-4c
Modules 1-4d
Module 1+5
Module 1.1 +5
Modules 1-4a +5
Modules 1-4b +5
Modules 1-4c +5
Modules 1-4d +5

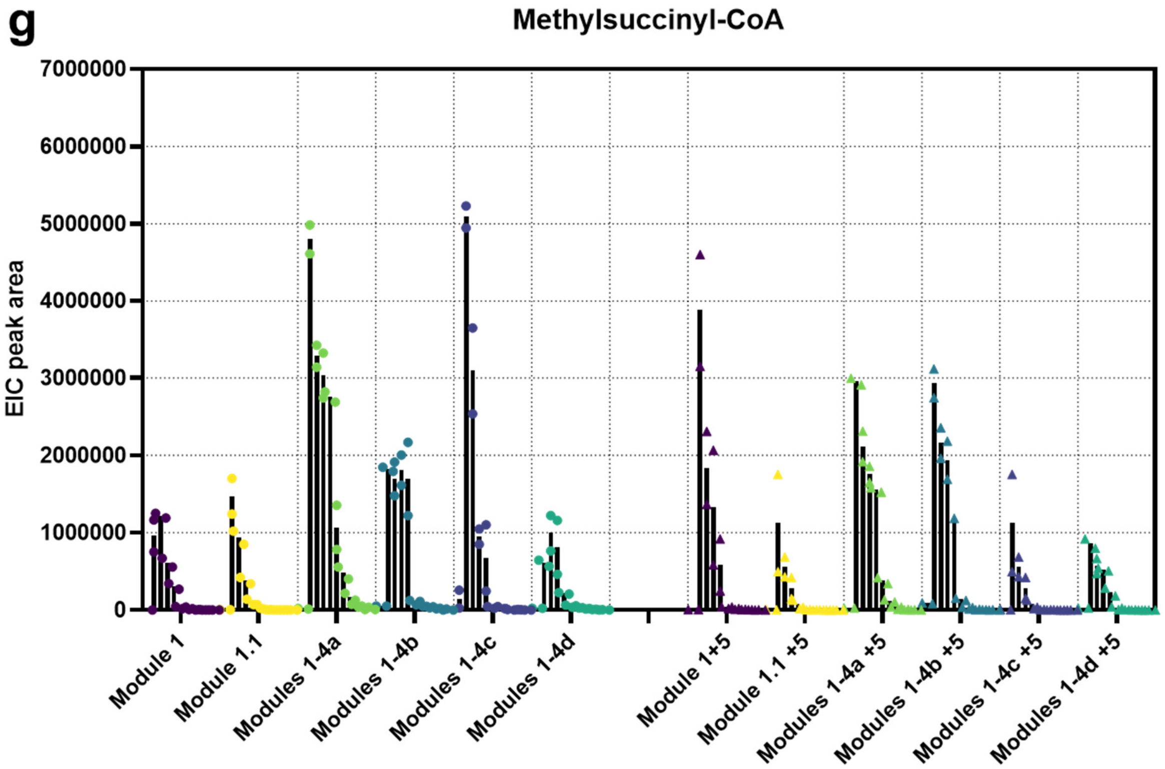
g
Methylsuccinyl-CoA
EIC peak area
7000000
6000000
5000000
4000000
3000000
2000000
1000000
0
Module 1
Module 1.1
Modules 1-4a
Modules 1-4b
Modules 1-4c
Modules 1-4d
Module 1+5
Module 1.1 +5
Modules 1-4a +5
Modules 1-4b +5
Modules 1-4c +5
Modules 1-4d +5

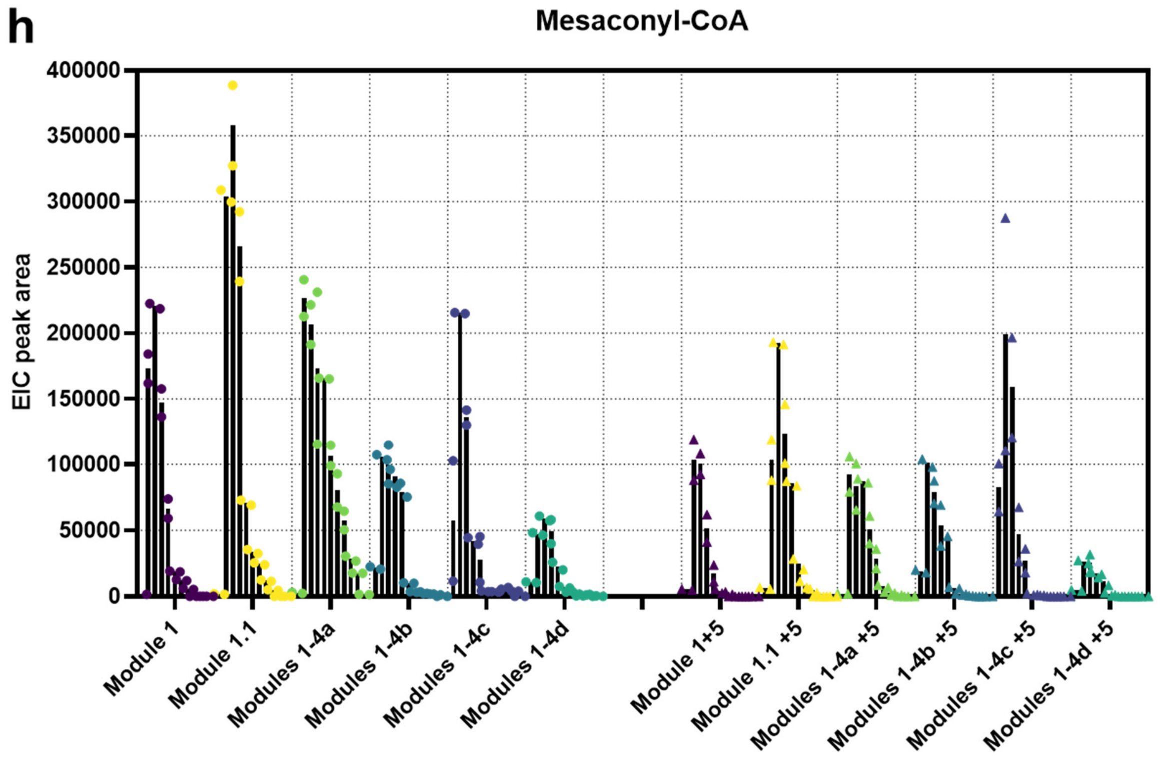
h
Mesaconyl-CoA
EIC peak area
400000
350000
300000
250000
200000
150000
100000
50000
0
Module 1
Module 1.1
Modules 1-4a
Modules 1-4b
Modules 1-4c
Modules 1-4d
Module 1+5
Module 1.1 +5
Modules 1-4a +5
Modules 1-4b +5
Modules 1-4c +5
Modules 1-4d +5

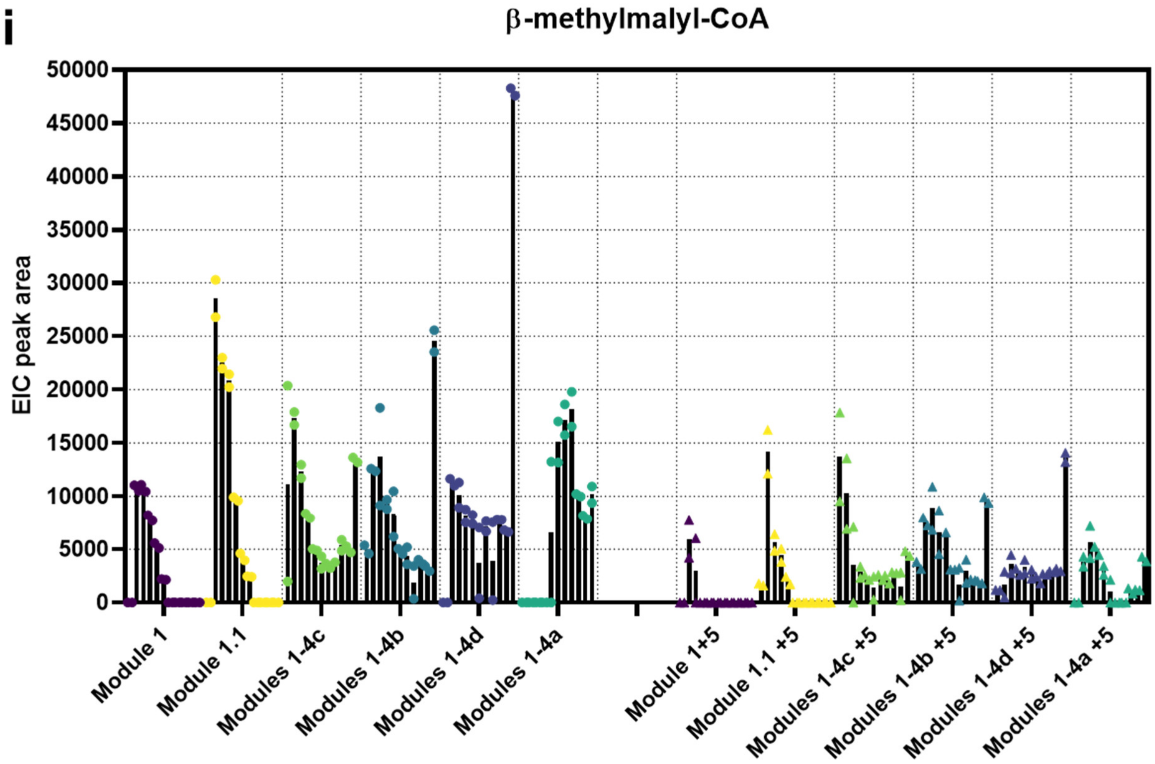

Supplementary Fig. 4. Extracted Ion Counts (EIC) peak areas of CoA intermediates of module 1. All shown values are the peak areas of the EICs of the quantifiers (see Table S5). The vertical lines separate the different assays as labelled on the x-axis. The bars represent the timepoints 0, 0.5, 1, 1.5, 2, 4, 6, 8, 10, 12 and 24 h (from left to right) in each assay. **a** Propionyl-CoA), **b** Methylmalonyl-CoA, **c** Succinyl-CoA, **d** γ-hydroxybutyryl-CoA, **e** Crotonyl-CoA, **f** Ethylmalonyl-CoA, **g** Methylsuccinyl-CoA, **h** Mesaconyl-CoA, **i** β-methylmalyl-CoA. The data was collected from two different experiments. The EICs of module 1, module 1.1, module 1+5 and module 1.1+5 were normalized by re-measuring samples from the initial dataset.

References

1. Schwander, T., Schada von Borzyskowski, L., Burgener, S., Cortina, N.S. & Erb, T.J. A synthetic pathway for the fixation of carbon dioxide in vitro. *Science* **354**, 900-904 (2016).
2. von Borzyskowski, L.S. et al. Marine Proteobacteria metabolize glycolate via the β-hydroxyaspartate cycle. *Nature* **575**, 500-504 (2019).
3. Sundaram, S. et al. A modular in vitro platform for the production of terpenes and polyketides from CO2. *Angewandte Chemie International Edition* (2021).
4. Kitagawa, M. et al. Complete set of ORF clones of Escherichia coli ASKA library (a complete set of E. coli K-12 ORF archive): unique resources for biological research. *DNA Res* **12**, 291-9 (2005).
5. Mracek, J., Snyder, S.J., Chavez, U.B. & Turrens, J.F. A soluble fumarate reductase in Trypanosoma brucei procyclic trypomastigotes. *J Protozool* **38**, 554-8 (1991).
6. Schwander, T. The design and realization of synthetic pathways for the fixation of carbon dioxide in vitro, Philipps-Universität Marburg (2018).
7. Hugler, M., Menendez, C., Schagger, H. & Fuchs, G. Malonyl-coenzyme A reductase from Chloroflexus aurantiacus, a key enzyme of the 3-hydroxypropionate cycle for autotrophic CO(2) fixation. *J Bacteriol* **184**, 2404-10 (2002).
8. Bernhardsgrütter, I. et al. The multicatalytic compartment of propionyl-CoA synthase sequesters a toxic metabolite. *Nature chemical biology* **14**, 1127-1132 (2018).
9. Opgenorth, P.H., Korman, T.P. & Bowie, J.U. A synthetic biochemistry molecular purge valve module that maintains redox balance. *Nat Commun* **5**, 4113 (2014).
10. Egloff, K. Engineering of an artificial autotrophic CO2 fixation pathway in Methylobacterium extorquens. (Philipps-Universität Marburg, 2014).
11. Lowry, B. et al. In vitro reconstitution and analysis of the 6-deoxyerythronolide B synthase. *Journal of the American Chemical Society* **135**, 16809-16812 (2013).
12. Erb, T.J., Retey, J., Fuchs, G. & Alber, B.E. Ethylmalonyl-CoA mutase from Rhodobacter sphaeroides defines a new subclade of coenzyme B12-dependent acyl-CoA mutases. *J Biol Chem* **283**, 32283-93 (2008).
13. Erb, T.J. The Ethylmalonyl-CoA Pathway, University of Freiburg (2009).
14. Söhling, B. & Gottschalk, G. Purification and characterization of a coenzyme-A-dependent succinate-semialdehyde dehydrogenase from *Clostridium kluyveri*. *Eur. J. Biochem.* **212**, 121-127 (1993).
15. Konneke, M. et al. Ammonia-oxidizing archaea use the most energy-efficient aerobic pathway for CO2 fixation. *Proc Natl Acad Sci U S A* **111**, 8239-44 (2014).
16. Zarzycki, J. et al. Mesaconyl-coenzyme A hydratase, a new enzyme of two central carbon metabolic pathways in bacteria. *J Bacteriol* **190**, 1366-74 (2008).
17. Erb, T.J., Frerichs-Revermann, L., Fuchs, G. & Alber, B.E. The apparent malate synthase activity of Rhodobacter sphaeroides is due to two paralogous enzymes, (3S)-Malyl-coenzyme A (CoA)/{beta}-methylmalyl-CoA lyase and (3S)- Malyl-CoA thioesterase. *J Bacteriol* **192**, 1249-58 (2010).
18. Sevinc, M.S., Ens, W. & Loewen, P.C. The cysteines of catalase HPII of Escherichia coli, including Cys438 which is blocked, do not have a catalytic role. *Eur J Biochem* **230**, 127-32 (1995).
19. Hoelsch, K., Suhrer, I., Heusel, M. & Weuster-Botz, D. Engineering of formate dehydrogenase: synergistic effect of mutations affecting cofactor specificity and chemical stability. *Appl Microbiol Biotechnol* **97**, 2473-81 (2013).

20. Nocek, B. et al. Polyphosphate-dependent synthesis of ATP and ADP by the family-2 polyphosphate kinases in bacteria. *Proceedings of the National Academy of Sciences* **105**, 17730-17735 (2008).
21. Scheffen, M. et al. A new-to-nature carboxylation module to improve natural and synthetic CO2 fixation. *Nature Catalysis* **4**, 105-115 (2021).
22. Schada von Borzyskowski, L. et al. Marine Proteobacteria metabolize glycolate via the beta-hydroxyaspartate cycle. *Nature* **575**, 500-504 (2019).
23. Rommel, T.O., Hund, H.K., Speth, A.R. & Lingens, F. Purification and N-terminal amino-acid sequences of bacterial malate dehydrogenases from six actinomycetales strains and from Phenylobacterium immobile, strain E. *Biol Chem Hoppe Seyler* **370**, 763-8 (1989).
24. Ueda, Y., Yumoto, N., Tokushige, M., Fukui, K. & Ohya-Nishiguchi, H. Purification and characterization of two types of fumarase from Escherichia coli. *J Biochem* **109**, 728-33 (1991).
25. Nolte, J.C. et al. Novel characteristics of succinate coenzyme A (Succinate-CoA) ligases: conversion of malate to malyl-CoA and CoA-thioester formation of succinate analogues in vitro. *Appl Environ Microbiol* **80**, 166-76 (2014).
26. Ito, S., Koyama, N. & Osanai, T. Citrate synthase from Synechocystis is a distinct class of bacterial citrate synthase. *Sci Rep* **9**, 6038 (2019).
27. Jordan, P.A., Tang, Y., Bradbury, A.J., Thomson, A.J. & Guest, J.R. Biochemical and spectroscopic characterization of Escherichia coli aconitases (AcnA and AcnB). *Biochem J* **344 Pt 3**, 739-46 (1999).
28. MacKintosh, C. & Nimmo, H.G. Purification and regulatory properties of isocitrate lyase from Escherichia coli ML308. *Biochem J* **250**, 25-31 (1988).
29. Schwander, T. The design and realization of synthetic pathways for the fixation of carbon dioxide in vitro, Philipps University Marburg (2017).
30. Bernhardsgrutter, I. et al. The multicatalytic compartment of propionyl-CoA synthase sequesters a toxic metabolite. *Nat Chem Biol* **14**, 1127-1132 (2018).
31. Saint Girons, I. et al. Structural and catalytic characteristics of Escherichia coli adenylate kinase. *J Biol Chem* **262**, 622-9 (1987).
32. Oeding, V. & Schlegel, H.G. Beta-ketothiolase from Hydrogenomonas eutropha H16 and its significance in the regulation of poly-beta-hydroxybutyrate metabolism. *Biochem J* **134**, 239-48 (1973).
33. Liu, Q., Ouyang, S.P., Chung, A., Wu, Q. & Chen, G.Q. Microbial production of R-3-hydroxybutyric acid by recombinant E. coli harboring genes of phbA, phbB, and tesB. *Appl Microbiol Biotechnol* **76**, 811-8 (2007).
34. Flamholz, A., Noor, E., Bar-Even, A. & Milo, R. eQuilibrator—the biochemical thermodynamics calculator. *Nucleic acids research* **40**, D770-D775 (2012).
